# Economic Statistical Design of Integrated X-bar-S Control Chart with Preventive Maintenance and General Failure Distribution

**DOI:** 10.1371/journal.pone.0059039

**Published:** 2013-03-18

**Authors:** Santiago Omar Caballero Morales

**Affiliations:** Technological University of the Mixteca, Huajuapan de Leon, Oaxaca, Mexico; University of East Piedmont, Italy

## Abstract

The application of Preventive Maintenance (PM) and Statistical Process Control (SPC) are important practices to achieve high product quality, small frequency of failures, and cost reduction in a production process. However there are some points that have not been explored in depth about its joint application. First, most SPC is performed with the X-bar control chart which does not fully consider the variability of the production process. Second, many studies of design of control charts consider just the economic aspect while statistical restrictions must be considered to achieve charts with low probabilities of false detection of failures. Third, the effect of PM on processes with different failure probability distributions has not been studied. Hence, this paper covers these points, presenting the Economic Statistical Design (ESD) of joint X-bar-S control charts with a cost model that integrates PM with general failure distribution. Experiments showed statistically significant reductions in costs when PM is performed on processes with high failure rates and reductions in the sampling frequency of units for testing under SPC.

## Introduction

Control charts are tools of Statistical Process Control (SPC) that monitor the state of a production process, identifying when the quality attributes of a product change. The concept of “control” is related to the quality attribute that is within specified limits (control limits) to ensure production stability and quality of products. If the attribute (i.e., weight, length, dimensions, etc.) is not within these limits, then the process is in an “out-of-control” state. In such case, is necessary to find and correct the assignable cause that originated this state (failure).

A control chart is defined by three main parameters: the size of the sample (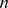), the sampling interval between samples (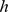), and the coefficient of the control limits (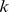). These parameters are selected based on economic and statistical restrictions because there are costs and times associated with sampling and searching of assignable causes: high sampling frequency would take more time from the process cycle time, and depending on the nature of the item, product loss. Also, close control limits would increase the frequency of failure alarms and rejection of products which not necessarily would be of low quality. The chart parameters must be selected following a methodology in order to minimize the “cost of quality” [1].

The Economic Design (ED) of control charts (the estimation of the parameters) considers the costs (in time and money) associated with sampling and searching/repairing of assignable causes. On the other hand, the Economic Statistical Design (ESD) additionally considers the statistical requirements, such as the probabilities of error Type I (detecting an out-of-control state when the process is fine) and II (not detecting an out-of-control state when the process is not fine) in the estimation of the parameters.

The ED of control charts was introduced in 1956 by Duncan [2] for X-bar (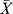) charts that monitor the mean of the quality characteristic of produced items. It had the following assumptions: the failure mechanism of the process had an Exponential probability distribution, there was only one assignable cause, and the sampling interval was constant. Other works extended the ED to ESD and covered other control charts: 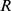, 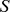, and 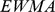 control charts were proposed to monitor variability [3]–[7]; 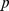 and 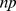 control charts were proposed to monitor proportion or number of nonconforming units within samples [8].

Variability is an important factor to control in a process because raw material, operators skills, machine calibration, etc., increase variability without affecting the process mean [9]. To keep control in both the mean and variability of a process the 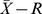 control chart has been used, although the 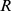 chart loses reliability when 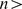
10 [10]. In this case the 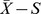 or 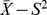 control charts are more suitable. Collani, Sheil [9], and Yang [7] proposed the ESD of 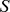 charts, considering the importance of the error Type I and II for minimization of costs. The ED and ESD of 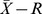 and 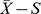 control charts was proposed by Davis and Saniga [6], [11], [12], pointing out the importance of controlling the mean and variance of the process. However in these cases, it was assumed that the sampling intervals were constant and that the process failed with an Exponential distribution.

An extension of these works was presented by Chiu [13] who considered the importance of Preventive Maintenance (PM) in the ED of 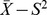 control charts to reduce long-term variability and failures that are only evident when the process reaches an out-of-control state. In [14] the ED of an 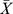 control chart combined with an age-replacement PM policy was presented. It was observed that reduction in operating costs was superior to the reduction achieved by using only the control chart or the PM policy. The relationship between SPC and PM has been recognized in other studies as in [15]–[20] identifying a link between equipment maintenance and product quality: “*Equipment maintenance*, *either corrective or preventive in nature*, *has a direct impact on the reliability of the equipment*, *and thus the performance of the equipment*. *Under the assumption that the equipment is used to manufacture some type of product*, *with improved performance of the equipment comes increased product quality*” [1].

This paper extends on the application of SPC with PM as some points were not completely covered by previous studies. First, most SPC is performed with the 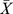 control chart which does not consider the variability of the production process [1], [14], [15], [20]–[23]. Second, many studies of design of control charts consider just the economic aspect while statistical restrictions must be considered to achieve charts with low probabilities of false detection of failures [14]–[16], [19], [20]. Third, the effect of PM on processes with different failure probability distributions has not been explored as most of the studies consider one distribution (i.e., Exponential [18], [19], [23] or Weibull [20], [24]).

Hence, this paper presents the Economic Statistical Design (ESD) of joint 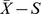 control charts to monitor mean and variability in a production process. In addition, the cost model integrates PM with general failure distribution (cases with Exponential, Gamma, and Weibull distributions are presented) and constant and variable sampling intervals. Experiments showed that PM decreases costs for processes with high failure rates and reduces the sampling frequency of units for testing under SPC.

### Background

#### Reliability Function

Consider that a cumulative distribution function 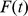 represents the probability that a unit, randomly taken from a population, will fail at most in time 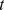 [25]. Now consider that, instead of taking one unit, 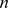 units are taken at the end of a time interval 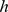. If it is of particular interest to get the distribution of the survival of the process, then the cumulative distribution function 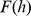 can be defined as the probability of the process failing (changing to an out-of-control state) at the end of the sampling interval 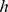.

Because the reliability (or survival) function of the process, 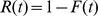, represents the probability that a unit will be working beyond time 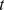 [25], the probability that a process will be working properly (in-control state) after the sampling interval 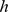 can be expressed as 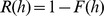.

Hence, the following probabilities are associated with the control states of a process:
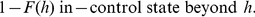
(1)

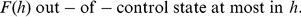
(2)


#### Detection of States: Significance Level and Power

The Significance Level 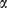 is the probability of the error Type I (false positive), which is the detection of an out-of-control state when the true state is in-control. Thus, if the null hypothesis 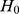  =  process is in-control state:
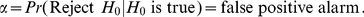
(3)

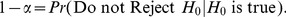
(4)


The probability of the error Type II (false negative), represented as 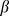, consists in the null detection of the out-of-control state when the process is truly in out-of-control state. Using Eq. 3 and 4 as reference:
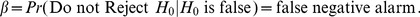
(5)

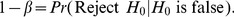
(6)1-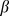 is also known as the Statistical Power of the control chart, which represents the ability of the chart to detect the out-of-control state when the process is indeed in such state. Thus, the levels of 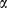 and 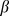 must be low and controlled when designing the control chart.

#### Significance Level and Power for 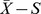 Control Charts

Although the cost model of a process may be used with different control charts, the definitions of 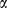 and 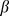 are dependent of the control chart. For the 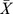 control chart, 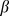 in terms of the control limits (Upper Control Limit UCL, Lower Control Limit LCL), is expressed as:
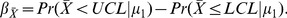
(7)where the random variable of interest is 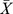 with N(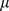, 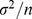) distribution. If 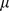 and 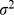 are known, the control limits are expressed as:
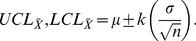
(8)and, if 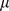 and 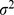 are unknown, these can be estimated from the 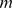 samples of size 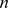 as:
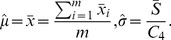
(9)giving the following control limits:
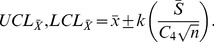
(10)In Eq. 9 and 10, 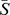 is the mean standard deviation of the 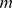 samples, and 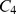 is a constant that depends on the size of the sample (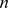). Note that 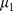 in Eq. 7 is the value that represents the change in the mean of the process, which is equal to 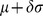, where 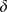 is the magnitude of that change. Hence, the error Type II probability of Eq. 7 can be expressed as:
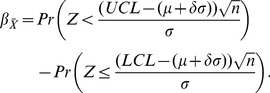
(11)

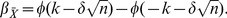
(12)


The error Type I for the 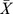 chart, 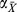 can be expressed as:
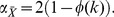
(13)


For the 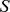 control chart, the Power in terms of the control limits can be expressed as:
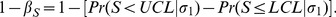
(14)where the random variable is 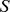 with 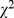 distribution with 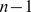 degrees of freedom, and 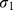 is the change in the standard deviation of the process (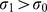, where 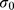 is the initial value). The control limits can be expressed in terms of the known standard deviation (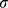), or an estimation of the same (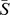), as follows:
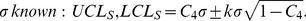
(15)

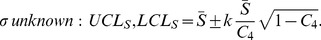
(16)


Commonly, 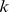 in Eq. 8, 10, 15, and 16 is restricted to 3 [26]. The relationship between the probability of the error Type II and the parameters of the 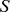 control chart then is expressed as:
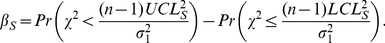
(17)


Similar formulations for 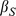 have been used by Saniga [6] and Collani [9]. The error Type I for the 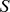 chart, 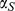, then can be expressed as:
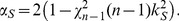
(18)


Finally, the joint error probabilities for the 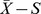 control chart are defined as:
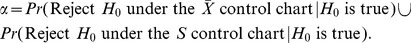
(19)

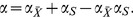
(20)

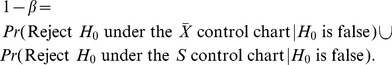
(21)

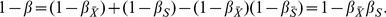
(22)

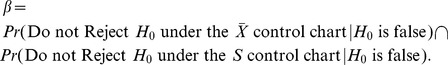
(23)

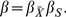
(24)


Note that for the 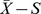 control chart two control limits coefficients are considered: 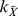 for the 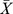 control chart, and 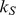 for the 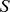 control chart. Also, because in the 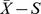 control chart two variables are monitored, two changes are considered: 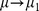, and 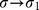. Hence, Eq. 12 is extended for the estimation of 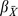 in Eq. 24 as follows:
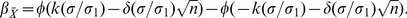
(25)


By integrating Eq. 1 and 2 with 20 and 24, the probabilities associated with the possible states of a process are obtained, and these are presented in Table 1.

**Table 1:** Control state probabilities.

State	Description	Probability	Expression
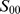	Process in-control state and no alarm	Pr(Process in-control)Pr(Do not Reject 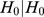 is true) = Pr( 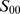 )	(1-F(h))(1- 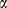 )
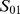	Process in-control state and false alarm	Pr(Process in-control)Pr(Reject 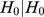 is true) = Pr( 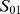 )	(1-F(h)) 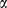
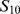	Process in out-of-control state and no alarm	Pr(Process out-of-control)Pr(Do not Reject 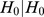 is false) = Pr( 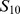 )	F(h) 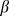
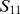	Process in out-of-control state with alarm	Pr(Process out-of-control)Pr(Reject 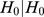 is false) = Pr( 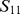 )	F(h)(1- 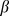 )

## Methods

### Base Cost Model with Constant Sampling Intervals

A production cycle is defined as the interval from the starting production time (in-control state) until the time when a change, caused by an assignable cause, occurs. This cycle includes the time required to detect and repair the assignable cause. Because a production cycle can be also defined as the time between successive in-control periods [27], the process can be considered as a serial of equally distributed independent cycles, a renewal process.

Under this assumption, the cost per cycle can be estimated as the accumulated cost from the beginning until the end of one cycle, and the average cost per unit of time can be estimated as the ratio of 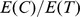, where 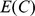 is the Expected Cost per Cycle and 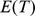 the Expected Cycle Length. The objective of the ESD is to minimize the costs per unit of time of a process: 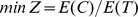 [21], [27], [28]. The Renewal Theory Approach proposed by Rahim and Banerjee [28] was presented as an alternative to obtain the equations for 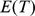 and 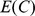 for Markovian and non-Markovian stochastic processes considering these assumptions.

A stochastic process has the Markov property if the conditional probability distribution of future states of the process depends only upon the present state, not on the sequence of events that preceded it. The Renewal Approach [28] studies the state of the system at the end of the first sampling interval. Depending upon the state of the system, the expected *residual* cycle length and cost can be computed. Then these values, together with the associated probabilities, define the renewal equations for 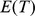 and 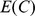.

The basic model studied by Duncan [2] had the Markov property and considered that a production cycle was integrated by the following components: (1) the in-control period; (2) the out-of-control period; (3) the time required to take a sample and interpret the results; and (4) the time needed to find the assignable cause. In [28] these components led to define the following states of the system at the end of the first sampling interval: (1) in-control state and no alarm; (2) in-control state and false alarm; (3) out-of-control and no alarm; and (4) out-of-control and true alarm. Then the equations for 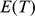 and 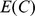 were obtained as the sum of the expected residual cycle length and cost multiplied by the probability associated with each of these states.

The expressions for 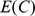 and 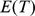 obtained with this approach in [28] were confirmed with those obtained with traditional approaches as that of Lorenzen and Vance [29] and Heikes *et al.* [30] for Markovian and non-Markovian models respectively. The approach also has been used to derive the equations of cost models with specific elements as that of Yang [27] which considered two assignable causes. This made the Renewal Approach suitable for the development of the cost models presented in this paper that are adaptations of the model of Rahim and Banerjee [28] which considered Exponential failure distribution and constant sampling intervals for the ED of 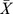 control charts.

The adapted base cost model under the Renewal Theory Approach assumes the following issues about the process:

1. The process starts in a stable in-control state with mean 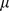 and variance 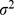. The event of an assignable cause changes the variance of the process from 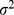 to 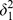 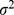, where 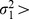
1 is the magnitude of the change and is known.
2. When a data point of the control chart is outside the control limits an alarm is generated, then the process is stopped and the search and repairing of the assignable cause starts. After the assignable cause is repaired the process returns to the in-control state, starting a new production cycle. The process is stopped also when there is a false alarm.
3. There is only one assignable cause and the process does not self-repair.
4. The time between failures has a general distribution.
5. The states of the system at the end of the first sampling interval are identified as: (1) 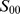 - in-control state and no alarm; (2) 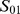 - in-control state and false alarm; (3) 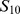 - out-of-control and no alarm; and (4) 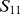 - out-of-control and true alarm. The probabilities associated with each state are presented in Table 1 and the details of the expected residual cycle length and cost associated with each state are presented in the following sections.

### Renewal Equations of the Expected Cycle Length 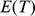

**Figure 1:**
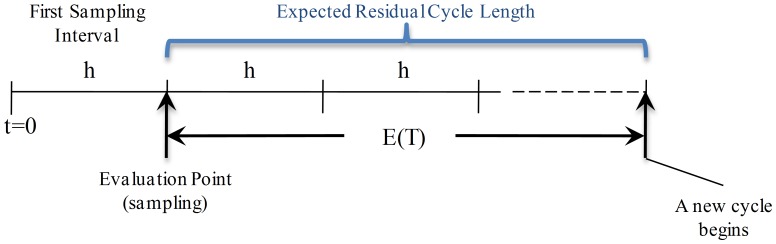
Expected Residual Cycle Length for State 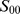. The process is in-control state and there is no alarm. Because the process is evaluated at the end of the first sampling interval 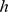 and no other events are associated with this scenario, the expected cycle length is 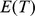.

**Figure 2:**
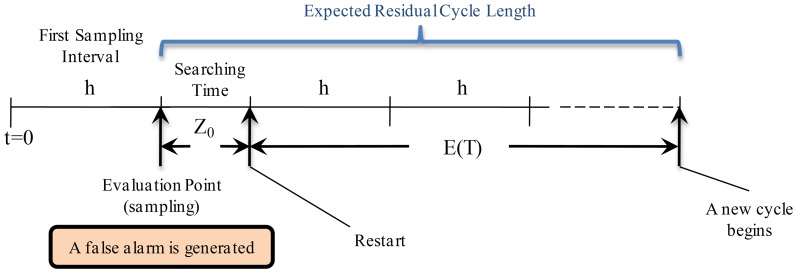
Expected Residual Cycle Length for State 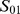. The process is in-control state but there is a false out-of-control alarm which causes the process to be stopped. This involves a time 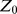 required to search for an assignable cause. After 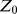 the process is restarted and the expected residual cycle length is equal to 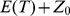.

**Figure 3:**
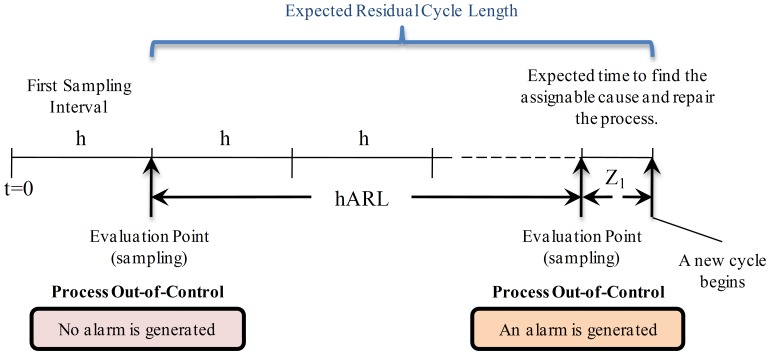
Expected Residual Cycle Length for State 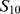. The process is in out-of-control state and there is no alarm (no detection). The necessary time to detect the failure can be expressed in terms of the number of samples before the alarm is generated. This number is a geometric random variable with mean 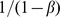 which is known as the Average Run Length (ARL). Thus, the necessary time to detect the out-of-control state is 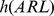 or 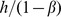. When the out-of-control is detected, the procedure to find the assignable cause and restore the process to an in-control state is performed with a time 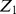. The expected residual cycle length is equal to 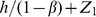.

**Figure 4:**
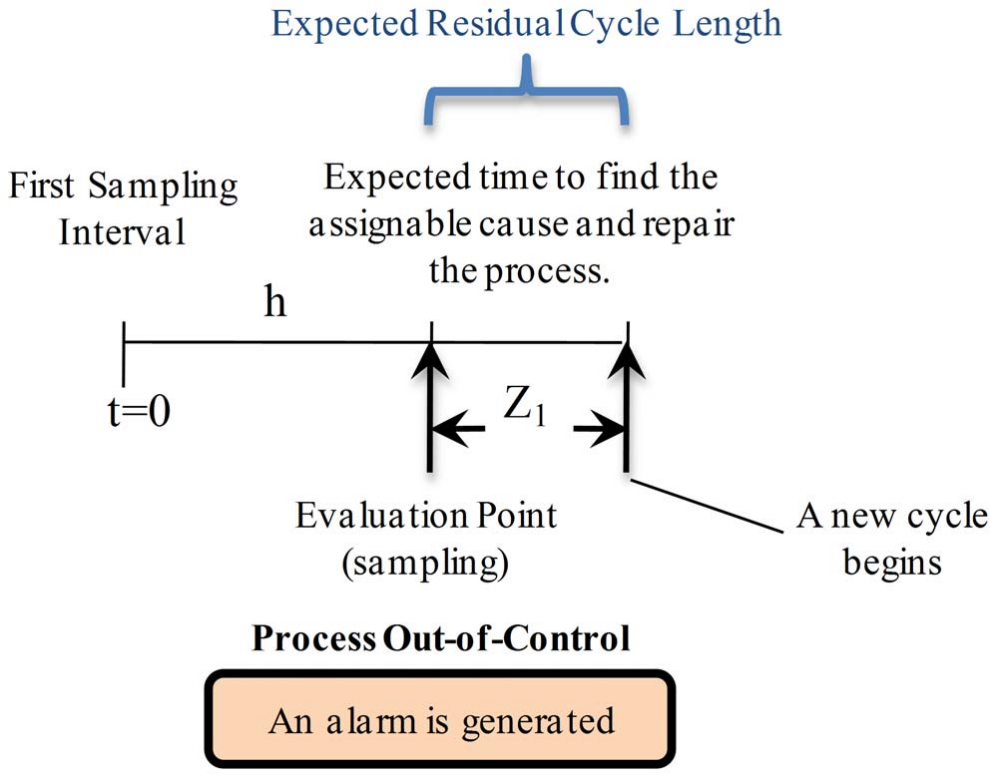
Expected Residual Cycle Length for State 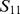. The process is in out-of-control state and detection is performed at the end of the interval where the process changed to this state. The only action that has to be performed is to find the assignable cause and restore the process. This only requires a time 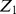 which also represents the expected residual cycle length.

- **State** 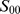: the state of the process is evaluated at the end of the first sampling interval 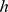, and depending on this the expected residual cycle length is estimated. As shown in Figure 1, in this case the process is in-control state with no alarm. Because there are no other events associated with this scenario, the expected residual cycle length is 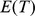.
- **State** 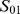: in this case there is a false out-of-control alarm which causes the process to be stopped, an action that involves loss of time and money. This scenario is shown in Figure 2, where the variable 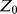 represents the time used to search the assignable cause when there is a false alarm. After that time the process is restarted and the expected residual cycle length is equal to 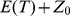 which considers the delay caused by the false alarm.
- **State** 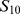: in this case the process is in out-of-control state and there is no alarm (no detection). Here it is important to consider the necessary time or intervals to detect the failure. Because each sampling interval is constant with length 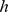, the necessary time to detect the failure can be expressed in terms of the number of samples before the alarm is generated. As show in Figure 3, this number is a geometric random variable with mean 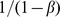, which is known as the *Average Run Length* (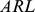) [3].
  Hence, the necessary time to detect the out-of-control state is 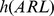, or 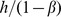. Observe that 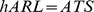 (*Average Time to Signal*), the average time to produce an alarm. When the out-of-control is detected, the procedure to find the assignable cause and restore the process to an in-control state is performed. In Figure 3, 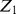 is the time associated with these tasks. When the process is restored a new cycle begins. Hence, the expected residual cycle length is equal to 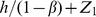.
- **State** 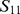: as shown in Figure 4, in this case the alarm is generated at the end of the interval where the process changed to the out-of-control state, thus there was a correct detection. In such scenario the only action that has to be performed is to find the assignable cause and restore the process, which only requires a time 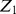. Hence, the expected residual cycle length is equal to 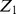.

The total 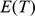 thus can be expressed as the sum of the expected residual cycle lengths, multiplied by their associated probabilities, of all states [27], [28]:
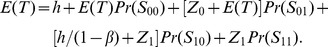
(26)

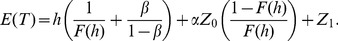
(27)


#### Renewal Equations of the Expected Cost


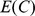
 **.** The process has the following associated costs:

- Sampling: always that a sample of size 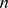 is taken, the cost 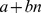 takes place, where 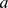 is the constant cost, and 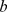 the variable cost of the sample.
- Producing in-control and out-of-control states: the cost per hour for producing in-control state is defined as 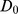 and the cost of producing in out-of-control state as 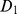 (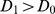).
- Searching and repairing: the cost of a false alarm is defined as 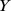, and the cost of searching and repairing an assignable cause is defined as 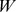.

**Figure 5:**
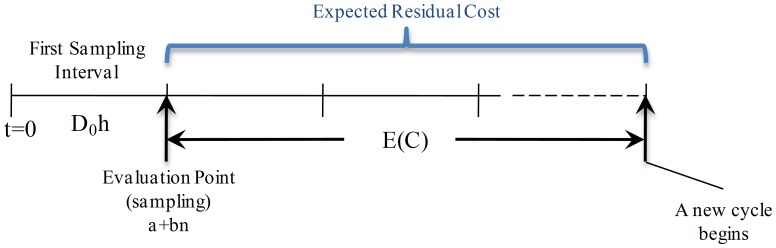
Expected Cost for State 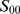. The process is in-control state and there is no alarm. Hence, only the costs associated with sampling (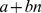) and producing in-control state in the first interval 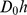 are considered. Thus, the cost consists of 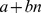 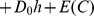, where 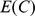 is the expected residual cost for this state.

**Figure 6:**
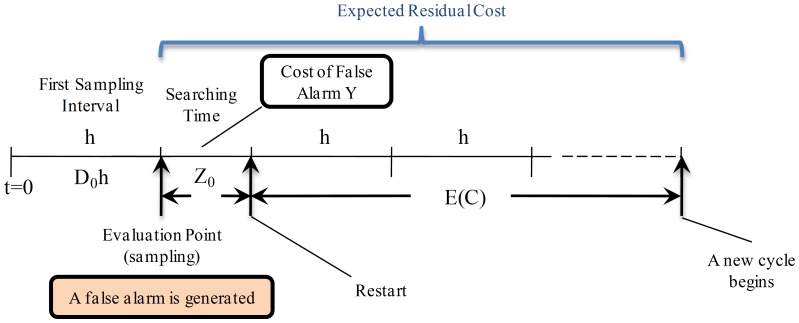
Expected Cost for State 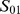. The process is in-control state but there is a false out-of-control alarm which causes the process to be stopped. Besides the sampling and in-control production costs, there is a cost associated with a false alarm (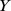) which implies losses because the process is stopped for a time 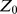. Thus, the expected cost for this state is 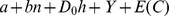, where 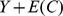 is the expected residual cost for this state.

**Figure 7:**
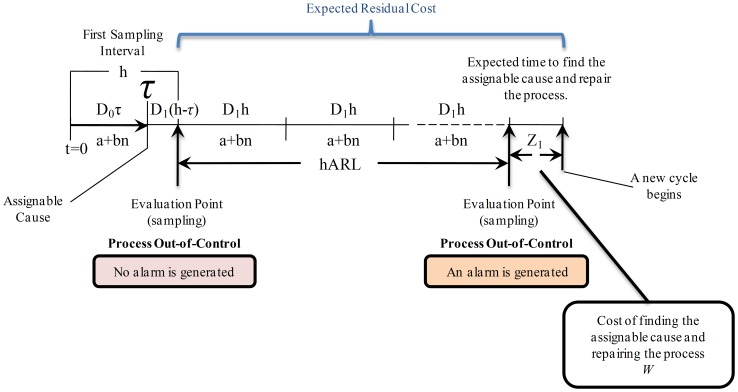
Expected Cost for State 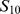. The process is in out-of-control state and there is no alarm (no detection). The assignable cause occurs within the first sampling interval at time 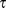 which changes the process to an out-of-control state. Thus, in the interval from 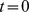 to 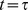 the process is in-control state, and from 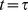 until 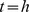 the process is in out-of-control state. Because of this, in the first sampling interval 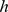 there are sampling costs (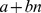), in-control production costs (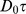), and out-of-control production costs (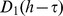). Then, sampling and out-of-control production costs take place while there is no detection (number of intervals estimated by 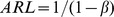). Finally, when detection is performed there is a cost 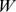 associated with interrupting the process, searching the assignable cause and repairing the process. Hence, the expected cost for this state is defined as 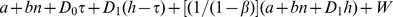, where 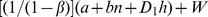 is the expected residual cost.

**Figure 8:**
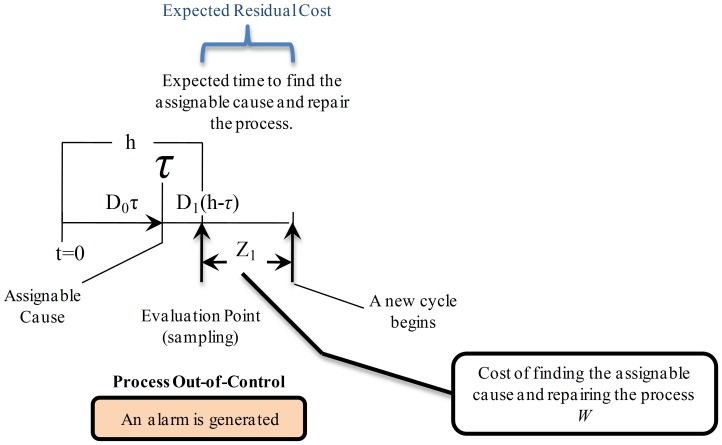
Expected Cost for State 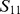. The process is in out-of-control state and detection is performed at the end of the interval where the process changed to this state. In addition to sampling costs (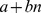), in-control production costs (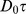), and out-of-control production costs (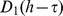) there is a cost 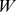 associated with interrupting the process, searching the assignable cause and repairing the process. Thus, the expected cost for this state is defined as 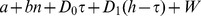, where 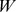 is the expected residual cost.

Considering these costs, the equations for 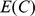 are derived as follows:

- **State** 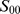: as presented in Figure 5, in this scenario only the costs associated with sampling and producing in-control state in the first interval 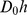 are considered. Thus, the expected cost is 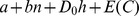, where 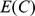 is the expected residual cost for this state.
- **State** 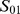: as shown in Figure 6, in this case besides the costs described above, there is a cost associated with a false alarm (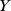), which implies losses because the process is stopped unnecessarily (for a time 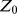). Thus, the expected cost for this state is 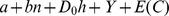, where 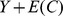 is the expected residual cost.
- **State** 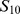: observe in Figure 7 that an assignable cause occurs within the first sampling interval in time 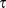 which changes the process to an out-of-control state. 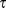 is a variable that was introduced by Duncan [2] for the case of the ED of a 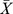-control chart when the failure mechanism had an Exponential distribution (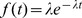, where 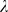 is the number of failures per unit of time). For a general 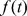, 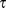 is defined as:
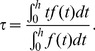
(28)
  In Figure 7 observe that, in the interval from 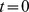 to 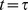 the process is in-control state, and that from 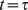 until 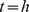 (the end of the interval 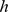) the process is in out-of-control state. Because of this, in the first sampling interval 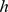 there are the following costs:
  Sampling cost: 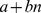;
  Cost for producing in-control state: 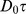;
  Cost for producing in out-of-control state: 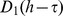.
  The evaluation of the process is performed at the end of the interval 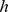 (sampling), however in this case the out-of-control state is not detected (there is no alarm). Hence, in the following intervals the process will continue producing in out-of-control state until the detection is successful, which happens after 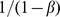 samples (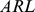). Meanwhile, during these intervals there are sampling costs (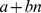) and losses for producing in out-of-control state (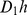). Thus, the cost of producing in out-of-control state until the detection takes place is given by 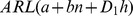.
  When detection is performed, the process is stopped and searching and repairing of the assignable cause is done with an associated cost 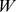. Finally, the expected cost for this state is defined as: 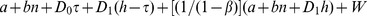, where 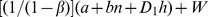 is the expected residual cost.
- **State** 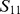: as presented in Figure 8, in this case the detection of the out-of-control state is performed successfully at the end of the first sampling interval 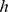, hence prompt procedures to find the assignable cause and restore the process are implemented with a cost 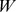. Thus, the expected cost for this scenario is 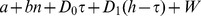, where 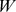 is the expected residual cost.

The total 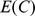 thus can be expressed as the sum of the expected costs, multiplied by their associated probabilities, of all states [27], [28]:
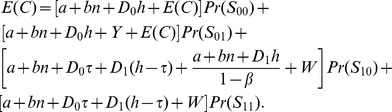
(29)

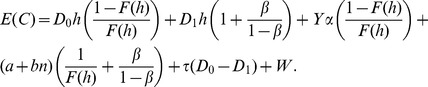
(30)


### Base Cost Model with Variable Sampling Intervals

In the model given by Eq. 27 and Eq. 30 when all sampling intervals are constant or fixed, 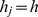 for all 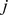 samples. When the sampling intervals are variable, 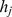 is different for each 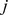 sample. In [21] Rahim *et al.* proposed to consider a specific number of 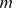 samples (sampling intervals) in the production cycle, 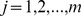, so the production cycle could be considered as truncated [31]. A truncated production cycle starts when a new component is installed and ends with a repair or after a fixed number of 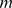 sampling intervals (at a given age 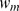). The cost model derived in this section is the model of Rahim and Banerjee [21] for general failure distribution and variable sampling intervals. The deduction was important to understand the model in order to develop the integrated cost model with PM.

The model makes the following assumptions:

1. The first interval 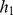 is randomly chosen.
2. The length of the next sampling intervals are chosen as 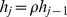, where 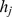 is the sampling interval for sample 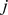, and 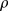 is a decrement factor. The sampling intervals 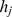 are computed by applying the decrement factor to the successor sampling interval, thus 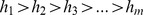, because as time continues the sampling frequency must increase given the natural wear and tear of the components of the process [21].
3. The number of sampling intervals is fixed and given (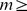 2).
4. The objective is to find 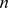, 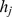 (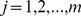), and 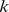 that minimize 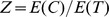.
5. There is an additional cost 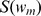 in 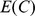 which is associated with the salvage cost of an equipment of age 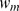.

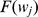
 is the cumulative distribution function of failure when the equipment (process) is of age 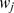, which is accumulated accordingly to the sampling over time. Hence, the age of a process at a given sampling interval 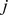 is given by:
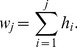
(31)
7. The failure probability (out-of-control probability) for a specific interval 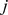 can be estimated as:
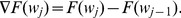
(32)

### Renewal Equations of the Expected Cycle Length 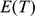

**Figure 9:**
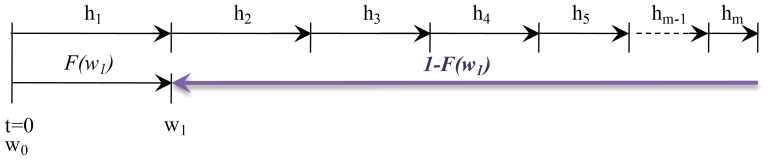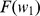 and 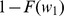 when the Sampling Interval is Variable. 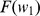
 represents the probability of being in out-of-control state at most in time 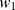, and 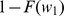 the probability of being in-control state from time 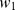. However this does not represent the probability of being in-control state in the interval 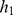. To include this interval, which starts in 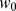 and ends in 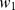, the corresponding probability must be 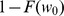. Hence, for the range of intervals from 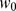 to 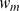, the following probabilities are defined for each sampling interval 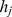: 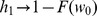; 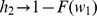;...; 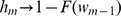.

- **State** 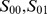: when the process is in-control state, the stop condition is given by (1) an alarm (true or false), or (2) by the age of the equipment ( =  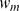). When there is no alarm at all, the stop condition is given only by 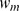. When the sampling intervals are variable, the probability to be in-control state cannot be generalized as 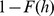 (Eq. 1), because each interval has an associated probability which is dependent on the age of the equipment (Eq. 32).
  In Figure 9, 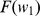 represents the probability of being in out-of-control state at most in time 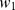, and 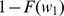 the probability of being in-control state from time 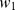. However this does not represent the probability of being in-control state in the interval 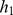. To include this interval, which starts in 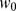 and ends in 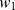, the corresponding probability must be 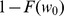. Hence, for the range of intervals from 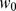 to 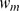, the following probabilities are defined for each sampling interval 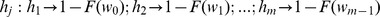.
  From these probabilities, the expected time when the process is in-control state and no alarm is generated (**State** 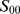) can be expressed as:
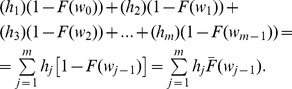
(33)
  The probability of a false alarm when the process is in-control state (**State** 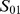) is represented by 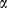 (Eq. 3), and is generated at the end of the first sampling interval. Because of this, it is not necessary to consider the in-control probability for this interval, and the in-control probability associated with other intervals can be expressed as:
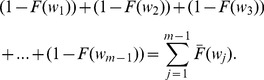
(34)
  Thus, the expected time to find an assignable cause when there is a false alarm (**State** 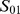) is expressed as:
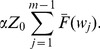
(35)
- **State** 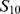, 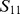: in **State** 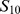 the process is already in out-of-control state (has failed), but there is no alarm. To derive the renewal equations some points must be considered:
  the interval where the process changed to the out-of-control state;
  the interval where the out-of-control state would be detected.
  Suppose that the process changes to the out-of-control state at some point within the interval 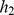 and it is not detected at the end of the same interval. By considering Eq. 32, the out-of-control probability in 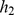 is given by 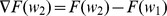. When the out-of-control probability is determined, it is necessary to consider the next intervals where detection can be performed (in this case, 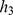, 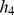, 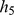,..., 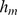). Thus, in general, if the assignable cause occurs in 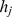, the detection can be performed in any interval 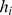 where 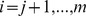. If detection is performed in 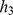 (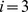), the no-detection probability can be expressed as 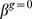, because the state was detected in the immediate following interval after the assignable cause occurred (thus there were 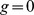 intervals with no detection). If however, detection takes place in interval 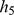 (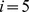), this means that the state was not detected in intervals 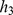 and 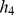 (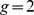), and thus there were two consecutive intervals where no detection was performed with probability 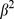. The index 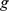 of 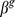 follows the sequence 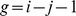, so in the case that the detection takes place until the end of the sampling intervals in 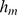, the probability of no detection would be 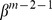. In general terms, the expected time to detect the out-of-control state can be expressed as 
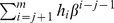
 for each interval 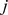 where an assignable cause occurs with a probability 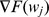.
  Thus, the expected time to detect the assignable cause when the process is in out-of-control state is given by:
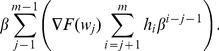
(36)
  In **State** 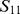 detection is successful at the end of the interval where the assignable cause occurred, thus the expected time consists of only 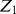.
  The total 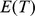 thus can be expressed as the sum of Eq. 33, 35, 36, and 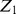:
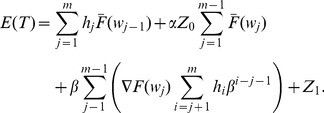
(37)

### Renewal Equations of the Expected Cost 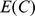

- **Costs of producing in-control states (** 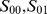 **)**: Eq. 33 provided the time that the process was in-control state with no false alarm (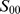). Because in Eq. 30 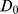 is the cost per hour of producing in-control state, then the expected cost of producing while the process is in-control state with no false alarm can be expressed as:
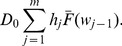
(38)When there is a false alarm (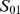) the process is stopped, and the expected time to search an assignable cause is given by Eq. 35. Because now it is required to consider the associated cost, 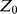 in Eq. 35 it can be replaced by the cost 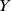 which corresponds to a false alarm:
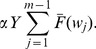
(39)
- **Costs of producing in out-of-control states (** 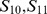 **)**: when there is a transition from the in-control to the out-of-control state, the following events are considered:
  The process is initially in-control state until the assignable cause occurred at some point within the sampling interval. As in the case of 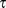 in Eq. 30, it is important to know the cost associated with the period of time in which the process was still in-control state. Because the process has a failure distribution given by 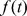, the mean expected probability for the interval of time from 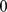 to 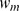 is:
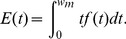
(40)
  Thus the cost:
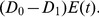
(41)represents the expected cost associated with the fraction of time within the interval in which the process is in-control.
  The process is in out-of-control state, and in this case, the costs depend on the age of the equipment 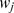 at the moment of the failure. As the age increases there will be intervals 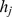 where the out-of-control probability will be more significant. Note that the process can change to an out-of-control state in any 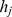 with a probability of 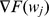. The associated cost of producing in out-of-control state can be expressed as:
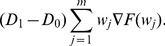
(42)
  As there is no detection of the out-of-control state, it is important to consider the cost associated with the intervals where no-detection is performed (the number of intervals until detection is successful). For this, Eq. 36 gives the time expected to detect the out-of-control state. Because during this time the process is in out-of-control state, the associated cost for this period can be expressed as:
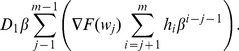
(43)
  Detection is successful and the repairing procedure starts. In this situation, the costs only consist of finding and repairing the assignable cause (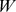).
- **Sampling Costs:** sampling is performed when the process is in-control state and while there is no detection (true alarm) of the out-of-control state. With this in mind, the first cost would be:
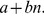
(44)which corresponds to the first sampling interval which is performed independently of the state of the interval. At the evaluation point of this interval a decision is made about continuing or not (in the case of a false alarm) with the process. For these in-control intervals the corresponding sampling costs are:
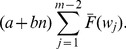
(45)
  Observe that 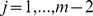, because the first and last intervals are not considered. The last one is not considered because there is already a stop condition given by 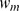.
  Now the associated costs of samples taken when the process is in out-of-control state and there is no alarm (no detection) are considered. Rahim *et al.* [21] defined this cost as 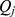, which is the expected number of samples taken after 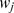 considering that the process is in out-of-control state from this time and there is no detection:
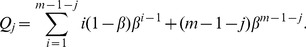
(46)
  As in Eq. 45, the first and the last intervals are not considered. Because there is no detection, it is necessary to consider the error Type II probability together with the out-of-control probability in the interval 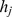 given by 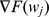. Hence, for each interval there is an associated cost 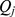, and the sampling cost when there is no detection is given by:
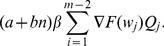
(47)
  The total sampling cost is then expressed as the sum of Eq. 44, 45, and 47:
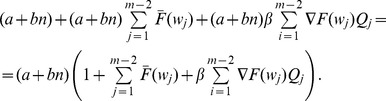
(48)
- **Salvage value for a machine of age** 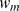: The model of Rahim *et al.* [21] considers a salvage value for the equipment used, allowing the possibility of replacement of the equipment depending on its age 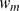 before a failure. This is only significant when the replacement produces an economic benefit. The salvage value for the equipment 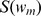 exists only when the process is in-control state within 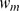, and so the corresponding cost during this period is:
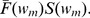
(49)Observe that this value represents a saving and not a cost.

The total Expected Cycle Cost 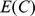 is expressed as the sum of all costs described in this section which are given by Eq. 38, 39, 41, 42, 43, 48, and 49:
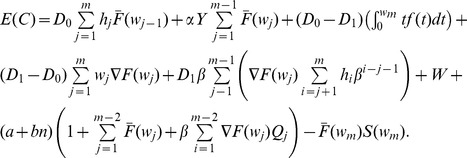
(50)


Eq. 37 and 50 match the model presented by Rahim *et al.* [21], which gives confidence about the deduction of the cost equations and hence, of the understanding of the cost function model to integrate the preventive maintenance.

### Integrated Cost Models with Preventive Maintenance

Preventive Maintenance (PM) has been proposed by diverse studies to increase the long-term reliability of equipment in a production process by reducing failure rates and age of the system [23], [32]. Chiu [13] integrated PM in a cost function for the Economic Design (ED) of 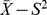 control charts assuming the following:

1. The process had increasing failure rate.
2. PM is performed at the evaluation point of constant sampling intervals. If the process is in-control state in time 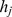, then PM is performed with an associated cost.

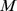
 includes costs associated with small adjustments or changes in machines or in other parts of the process (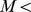 repairing cost).
4. PM does not restore the process from an out-of-control state to an in-control state.
5. The process is stopped when the PM is performed.

These assumptions were similar to those presented by more recent studies which also had significant additional considerations. In [15] Ben-Daya and Rahim also considered performing PM at the evaluation point of constant sampling intervals. However Chen *et al.* [23] stated that performing PM at each evaluation point would increase costs. As an alternative they proposed a “threshold” for the quality characteristic measured during a sampling interval to decide whether or not to perform PM. Rahim *et al.* [32] proposed that PM activities could be performed at 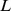 integer multiples of evaluation points, considering also that production ceases during PM.

Mehrafrooz and Noorossana [18] proposed different types of maintenance: Preventive, Corrective, Compensatory, and Planned maintenance. In their work, “true” out-of-control signals require Preventive maintenance while “false” alarms require Compensatory maintenance. Corrective maintenance is performed whenever process stops due to a failure, and Planned maintenance is the one scheduled to be performed after 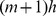 in-control intervals. However, a common assumption of some works (i.e., [18], [32]) is that PM is capable of restoring an equipment to a “good-as-new” condition, something that is not a realistic situation as discussed in [23]. Also, a single failure distribution is considered (i.e., Exponential [18], [23]) and thus, the effect of PM is not fully studied.

In this paper is assumed the following:

1. PM does not restore the process to a “good-as-new” condition although it decreases the failure rate after each implementation [32]. Failure rate was considered to be reduced by extending the period of time between failures. For this, a constant 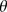 was defined as the possible gain in the life expectancy of the process and was integrated in the period of time between failures. It was considered to be at least of 10% of the original time between failures.
2. In terms of [18], Corrective maintenace is implicit in the activity of searching/repairing an assignable cause. PM is performed at each evaluation point while the process is detected to be in-control state (thus, Preventive 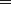 Compensatory 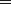 Planned maintenance).
3. The process has general failure distribution and the following are considered: Exponential, Weibull, and Gamma.
4. Sampling intervals are constant and variable.
5. The process can continue or be stopped while performing PM: 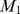 is the cost of PM if the process continues, and 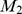 the cost of PM if the process is stopped (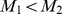).
6. Taking as reference the cost of repairing the process from an out-of-control state, the PM cost was set to 10% and 30% of 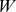 if the process continues while performing PM or if is stopped respectively.

Thus, the study of Chiu [13] about PM is extended for the ESD of 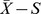 control charts with the more complex cost function model of Rahim *et al.* [21] for variable sampling intervals and general failure distributions. The work of Linderman [33] was also reviewed to allow, by means of a binary variable (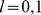), the modelling of the situation of performing PM without interrupting the process. Thus, a more comprehensive insight is presented about the effect of PM on the reliability of a process. In order to keep consistency with the base models of Rahim *et al.* [21], [28], the proposed models share the same terminology for 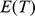 and 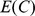.

Depending on the kind of process, if it is necessary to stop the process while performing PM (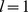), then a delay 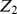 is added to each sampling interval if the process is in-control state. This is common in situations when some machine parts are worn-out and need to be replaced, or too much waste is accumulated in a machine. Another scenario that requires attention, independently if the process is stopped or not, is lubrication of mechanic parts, which can be performed with the process working without any delay 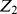, although it still implies a cost. Thus, 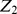 represents the expected time to perform PM. In the following sections the integration of these concepts is presented.

#### Constant Sampling Intervals

The modified Eq. 26 for the Expected Cycle Length 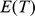 is:
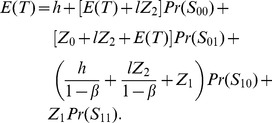
(51)


Note that, for state 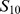, 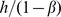 is the time required to detect that the process is in out-of-control state. Thus, 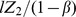 is defined as the time that PM was performed while the process was in out-of-control state before detection. Eq. 51 for 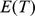 is reduced to the following expression:
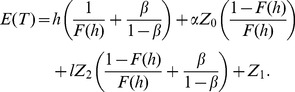
(52)


In similar way, the expression for the Expected Cost 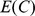 of Eq. 29 with PM is derived:
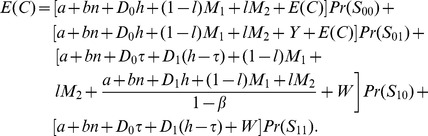
(53)

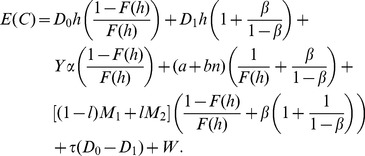
(54)


#### Variable Sampling Intervals

When the sampling intervals are constant, PM is performed at the end of the sampling interval 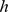 as long as the process is in-control state (or the out-of-control is not detected). For such case, a new sampling interval is established that includes the associated PM:
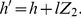
(55)


If the process is not interrupted (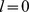) while performing the PM then there is no delay, hence 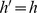 from the original cost model. Now, for variable sampling intervals, the same principle can be applied:
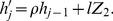
(56)


Because it is considered that PM is constant for each sampling interval, the Eq. 31 for 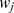 is adjusted as follows:
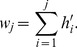
(57)


Thus, Eq. 37 for 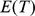 with PM is modified as:
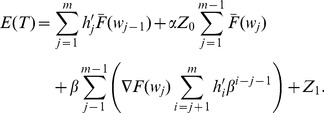
(58)


Eq. 50 for 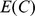 is modified when adding PM given by 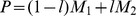:
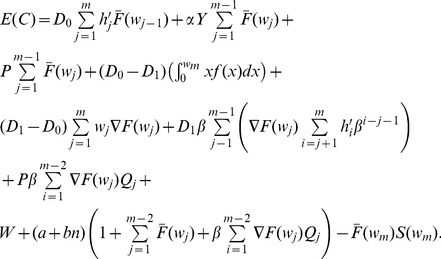
(59)where:

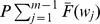
 is the cost of performing PM when the process is in-control state.

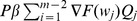
 is the cost of performing PM when the process is in out-of-control state and there is no detection. Hence, this cost depends on the number of samples taken while there is no true alarm.

## Results and Discussion

### Effect of PM on the Failure Distribution

It is expected that, as PM involves continuous adjustments and replacements of soon-to-be faulty parts, it would increase the reliability of the process in the long term. This could be reflected as a decrease in the number of failures in a given time period. Hence, this can have a direct effect on the life expectancy of the process, which is associated with the parameters of the failure distribution modelled by 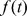.

In this paper three probability distributions are considered for the failure mechanism of the process: Exponential, Weibull, and Gamma, and the effect of PM on these distributions are presented in the following sections.

#### Exponential Distribution

For the Exponential distribution:
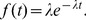
(60)where 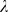 is the main parameter of the distribution and represents the known number of failures per unit of time. When PM is performed it is assumed that the life expectancy of the process can be increased, changing the length of the unit of time in which failures would occur.

It is considered that, by performing PM a gain 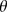 in the life expectancy is obtained. Additionally, if the process is stopped during the performance of PM (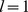) the associated delay 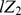 can be considered as another gain in the life expectancy of the process. Thus, the unit of time where failures would occur can be expressed as:
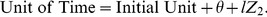
(61)


Hence, the adjusted parameter 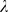 for the time between failures can be expressed as:
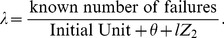
(62)


Because 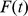 represents the probability that a unit selected randomly from a population will fail at most in time 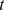, for the Exponential distribution 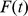 is expressed as:
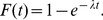
(63)


**Figure 10:**
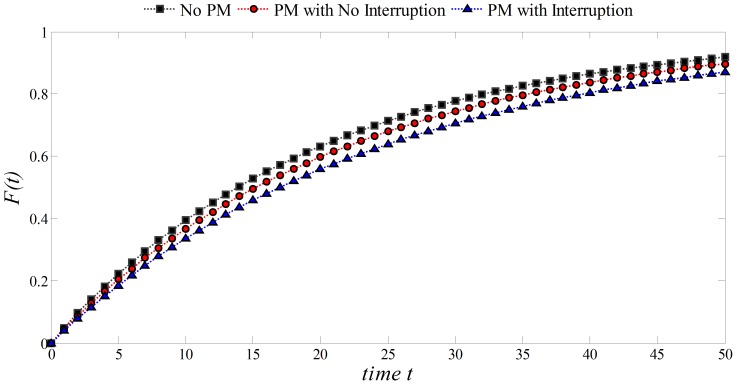
Effect of PM on the Exponential distribution: 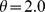, 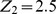, *Initial Unit* = 20. 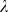
 is the main parameter of the distribution and represents the known number of failures per unit of time. When PM is performed a gain 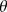 in the life expectancy can be obtained. This would increase the *Initial Unit* of time where failures are likely to take place. In addition, if the process is stopped during the performance of PM (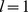) the associated delay 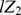 can be considered as another gain in the life expectancy of the process. Thus, the unit of time where failures would occur can be expressed as *Unit of Time* = *Initial Unit* + 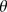 + 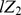. It is observed that for 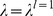 and 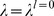 (PM with/without interruption of the process), the failure probabilities decreased at time 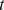. The lowest failure probability is accomplished when the process is stopped while performing PM, and the highest when there is no PM.

In Figure 10, it is observed that for 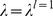 and 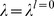 (PM with/without interruption of the process), the failure probabilities decreased at time 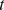. Thus a process with such patterns would be more reliable. Note that the lowest failure probability is accomplished when the process is stopped while performing PM, and the highest when there is no PM.

#### Weibull Distribution

For the Weibull distribution:
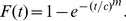
(64)where 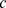 represents the time when the process is likely to fail, identifying in this way the life expectancy of the process. 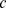 is known as the *scale parameter* and 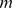 as the *form parameter*. Note that when 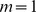 the Weibull distribution is approximated to the Exponential distribution with 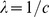.

Similar to Eq. 62 with PM, the time at which the system would fail is expressed as:
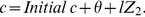
(65)


**Figure 11:**
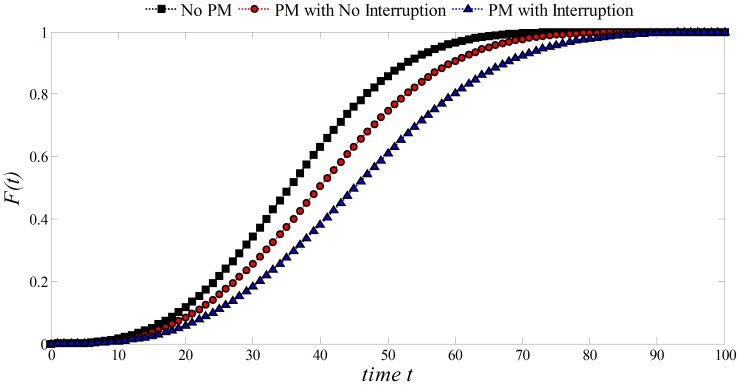
Effect of PM on the Weibull distribution: 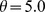, 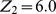, 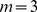, *Initial c*  = 40. 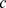
 represents the time where the process would fail, identifying in this way the life expectancy of the process. 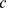 is known as the *scale parameter* and 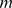 as the *form parameter*. When 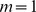 the Weibull distribution is approximated to the exponential distribution with 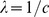. When PM is performed a gain 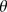 in the life expectancy can be obtained. This would increase the *Initial c*, and if the process is stopped during the performance of PM (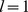), the associated delay 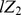 can be considered as another gain in the life expectancy of the process. Thus, the time at which the system would fail is expressed as: 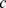 = *Initial c* 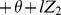. Although all cases achieve the same probability level by 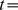
100, there is a marked delay when PM is performed. While in the original case with no PM the failure probability is 50% by 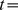
35, when PM is performed without interruption the probability at 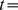
35 is 40% (50% is reached when 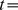
40). When there is PM with interruption, in 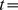
35 the failure probability is 27%, reaching 50% in 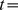
45.

As in the Exponential case, in Figure 11 the behavior of the Weibull failure distribution is shown when PM is performed. Although all cases achieve the same probability level by 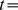
100, there is a marked delay when PM is performed. While in the original case with no PM the failure probability is 50% by 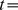
35, when PM is performed without interruption the probability at 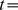
35 is 40% (50% is reached when 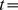
40). When there is PM with interruption, in 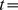
35 the failure probability is 27%, reaching 50% in 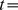
45.

For real purposes, 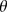 depends on the type of process, and it can be estimated from experiments performed to measure the strength or resistance of the system before and after the PM. In this work 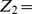
0.75 and 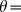
0.5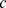 were used.

#### Gamma Distribution

For the Gamma distribution:
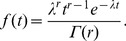
(66)where 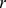 is termed as the *form parameter*, and 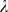 the *scale parameter*. For convenience, in this paper 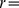
2 is used, which gives the following expression for 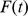:
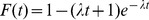
(67)



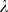
 is related to the life expectancy of the process which is considered to be increased PM by 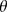 when PM is implemented. If PM is performed with interruption of the production process (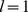) then the associated time to this task 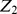 can be considered as another gain in the life expectancy. If 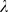 = 1/(*Unit of Time until Failure*) then:
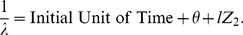
(68)


**Figure 12:**
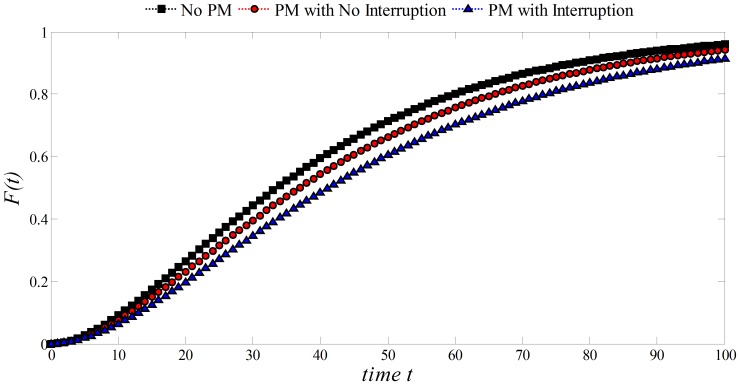
Effect of PM on the Gamma distribution: 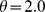, 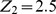, *Initial Unit* = 20. 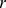
 is termed as the *form parameter*, and 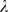 the *scale parameter*. For convenience in this work 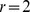 was used. 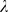 is related to the life expectancy of the process which is considered to be increased by 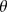 when PM is implemented. If PM is performed with interruption of the production process (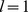) then the associated time to this task 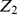 can be considered as another gain in the life expectancy. Because 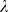  = *1*/(*Unit of Time until Failure*), then 1/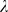  =  *Initial Unit of Time* + 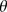 + 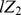. As presented, PM with interruption presents the lower probability of a failure for 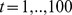. For example, for 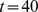, the failure probability is approximately of 48% and 53% for PM with 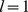 and 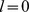 respectively. However if no PM is implemented, the failure probability is near 60%.

As presented in Figure 12, the failure distribution follows the same pattern as in the Exponential and Weibull cases. PM with interruption presents the lower probability of a failure for 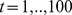. For example, for 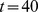, the failure probability is approximately of 48% and 53% for PM with 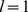 and 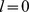 respectively. However if no PM is implemented, the failure probability is near 60%.

### Effect of the PM on the ESD of 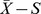 Control Charts

Matlab 2008b was used as the programming platform required to compute the cost models and perform the algorithm to achieve the ESD of the joint 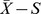 control chart. Diverse algorithms have been used to optimize the chart parameters for a given case. Among them, Genetic Algorithms (GAs) [8], [24], [31] and Tabu Search (TS) [32] have shown success for these tasks.

Previously a TS algorithm was developed to solve cost models for the ED and ESD of 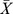, 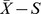 charts. This algorithm improved the ratio 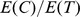 when compared with GAs, Hooke and Jeeves (HJ) and Combinatorial Methods (CB) as presented in [34]. Because the TS algorithm was validated with different cost models (Rahim *et al.* [21], [28], Ruvalcaba [31], Saniga *et al.* [6]), it was used for the optimization of the 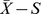 with PM presented in this work.

In Table 2 the data used for the ESD of the 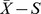 control chart with PM and constant/variable sampling intervals with Exponential, Weibull, and Gamma failure distributions is presented. The results, presented in Tables 3 and 4 were obtained with 20 iterations of the solving algorithm, and these are discussed in the following sections.

**Table 2:** Costs and Times for the ESD of 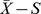 Control Charts with PM.

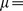 182	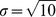	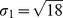	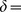 0.5
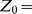 0.25	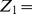 1	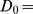 50	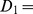 950
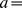 20	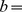 4.22	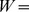 1100	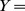 500
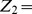 0.75	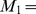 100	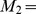 300	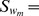 1100
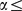 0.15	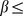 0.20	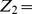 0.75	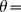 0.5 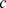

**Table 3:** Results of the Integrated Model with Constant Sampling Intervals.

Failure Distribution	Parameters	Models	*l*	*n*	*h*	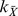	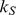	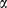	1- 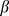	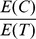	Reduction%
Exponencial	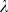 = 0.0505	BASE	-	33	2.96	2.00	2.16	0.0453	0.9639	215.22	-
		PM	0	35	4.73	1.81	1.48	0.0708	0.9821	207.20	3.73
		PM	1	37	4.81	1.86	1.42	0.0631	0.9856	212.82	1.12
Exponencial	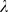 = 0.1010	BASE	-	32	2.20	1.94	2.27	0.0522	0.9597	294.50	-
		PM	0	36	3.23	1.85	1.41	0.0645	0.9843	278.38	5.47
		PM	1	37	3.44	1.89	1.44	0.0583	0.9848	271.99	7.64
Exponencial	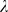 = 0.2525	BASE	-	27	1.45	1.72	2.71	0.0845	0.9324	443.31	-
		PM	0	32	2.28	1.62	1.39	0.1065	0.9818	413.19	6.79
		PM	1	34	2.26	1.74	1.34	0.0843	0.9840	372.04	16.08
Exponencial	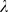 = 0.5050	BASE	-	25	1.16	1.65	2.31	0.0986	0.9400	589.03	-
		PM	0	34	1.75	1.74	1.34	0.0857	0.9842	549.33	6.74
		PM	1	34	1.56	1.71	1.35	0.0889	0.9844	459.86	21.93
Weibull, Form = 1	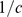 = 0.0505	BASE	-	42	2.85	2.56	1.48	0.0106	0.9814	225.54	-
		PM	0	42	4.45	2.56	1.48	0.0106	0.9814	208.81	7.42
		PM	1	32	4.73	2.03	1.43	0.0433	0.9715	209.32	7.19
Weibull, Form = 1	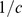 = 0.1010	BASE	-	31	2.03	2.10	1.36	0.0391	0.9690	295.09	-
		PM	0	30	3.23	2.00	1.46	0.0463	0.9653	272.03	7.81
		PM	1	31	3.46	2.09	1.42	0.0377	0.9673	266.83	9.58
Weibull, Form = 1	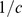 = 0.2525	BASE	-	30	1.67	2.00	1.34	0.0512	0.9693	451.28	-
		PM	0	30	2.47	2.00	1.34	0.0512	0.9693	407.84	9.63
		PM	1	30	1.80	2.00	1.38	0.0479	0.9678	364.81	19.16
Weibull, Form = 1	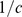 = 0.5050	BASE	-	30	1.14	1.99	1.42	0.0476	0.9669	601.23	-
		PM	0	30	1.94	1.99	1.42	0.0476	0.9669	543.37	9.62
		PM	1	31	1.15	2.01	1.46	0.0443	0.9680	450.98	24.99
Weibull, Form = 2	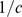 = 0.0505	BASE	-	31	4.45	2.00	1.47	0.0454	0.9680	115.69	-
		PM	0	31	6.85	2.00	1.47	0.0454	0.9680	113.51	1.88
		PM	1	31	7.93	2.01	1.48	0.0449	0.9675	125.72	−8.67
Weibull, Form = 2	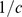 = 0.1010	BASE	-	30	2.52	1.99	1.58	0.0462	0.9612	160.68	-
		PM	0	30	4.12	1.99	1.38	0.0485	0.9680	154.79	3.67
		PM	1	32	4.68	2.11	1.46	0.0353	0.9687	166.50	−3.62
Weibull, Form = 2	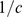 = 0.2525	BASE	-	33	1.50	2.03	1.46	0.0425	0.9734	283.55	-
		PM	0	33	2.30	2.03	1.46	0.0425	0.9734	260.62	8.09
		PM	1	32	2.36	2.11	1.55	0.0345	0.9654	246.61	13.03
Weibull, Form = 2	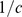 = 0.5050	BASE	-	31	0.98	2.09	1.52	0.0368	0.9637	436.52	-
		PM	0	30	1.38	1.99	1.42	0.0476	0.9668	391.61	10.29
		PM	1	32	1.42	2.11	1.48	0.0346	0.9677	323.39	25.92
Weibull, Form = 3	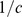 = 0.0505	BASE	-	32	5.79	2.10	1.44	0.0360	0.9693	90.26	-
		PM	0	32	9.79	2.10	1.44	0.0360	0.9693	89.99	0.30
		PM	1	31	10.76	2.09	1.38	0.0385	0.9685	100.69	−11.56
Weibull, Form = 3	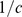 = 0.1010	BASE	-	31	3.20	2.10	1.47	0.0363	0.9653	121.02	-
		PM	0	30	5.47	1.99	1.41	0.0477	0.9673	119.38	1.36
		PM	1	35	6.41	2.26	1.42	0.0242	0.9742	133.88	−10.63
Weibull, Form = 3	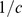 = 0.2525	BASE	-	30	1.48	2.00	1.47	0.0463	0.9651	206.16	-
		PM	0	31	2.68	2.10	1.57	0.0362	0.9619	198.57	3.68
		PM	1	30	2.92	1.99	1.37	0.0486	0.9682	199.79	3.09
Weibull, Form = 3	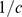 = 0.5050	BASE	-	33	1.00	2.12	1.43	0.0347	0.9721	343.86	-
		PM	0	32	1.44	2.02	1.53	0.0437	0.9689	317.39	7.70
		PM	1	33	1.86	2.04	1.47	0.0413	0.9729	272.22	20.83
Weibull, Form = 4	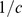 = 0.0505	BASE	-	31	7.39	2.09	1.45	0.0368	0.9660	79.48	-
		PM	0	31	10.92	2.01	1.44	0.0446	0.9686	80.22	−0.93
		PM	1	33	11.75	2.04	1.44	0.0421	0.9738	90.91	−14.38
Weibull, Form = 4	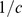 = 0.1010	BASE	-	33	3.98	2.12	1.54	0.0337	0.9686	105.77	-
		PM	0	33	6.38	2.12	1.54	0.0337	0.9686	104.81	0.91
		PM	1	30	7.42	1.99	1.53	0.0464	0.9630	117.17	−10.78
Weibull, Form = 4	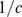 = 0.2525	BASE	-	30	1.95	1.99	1.60	0.0462	0.9606	174.90	-
		PM	0	30	2.75	1.99	1.60	0.0462	0.9606	170.37	2.59
		PM	1	30	3.46	1.99	1.41	0.0475	0.9672	176.56	−0.95
Weibull, Form = 4	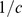 = 0.5050	BASE	-	31	1.00	2.01	1.52	0.0447	0.9663	283.50	-
		PM	0	32	1.64	2.03	1.40	0.0436	0.9724	273.84	3.41
		PM	1	31	1.99	2.10	1.46	0.0364	0.9657	239.94	15.37
Gamma, Form = 2	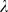 = 0.0505	BASE	-	38	6.55	1.94	1.54	0.0528	0.9839	102.47	-
		PM	0	41	10.40	2.01	1.42	0.0448	0.9887	100.31	2.11
		PM	1	43	11.59	2.06	1.39	0.0400	0.9906	109.96	−7.31
Gamma, Form = 2	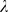 = 0.1010	BASE	-	35	3.75	1.81	1.40	0.0705	0.9836	136.06	-
		PM	0	37	6.68	1.90	1.37	0.0592	0.9859	130.29	4.24
		PM	1	37	7.24	1.90	1.45	0.0574	0.9844	139.95	−2.86
Gamma, orm = 2	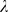 = 0.2525	BASE	-	33	2.43	1.72	1.53	0.0858	0.9790	219.17	-
		PM	0	36	3.55	1.79	1.40	0.0735	0.9855	203.55	7.13
		PM	1	33	3.83	1.72	1.34	0.0894	0.9827	200.98	8.30
Gamma, Form = 2	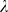 = 0.5050	BASE	-	30	1.59	1.60	1.56	0.1106	0.9742	328.46	-
		PM	0	33	2.43	1.72	1.37	0.0866	0.9821	296.76	9.65
		PM	1	33	2.73	1.73	1.33	0.0885	0.9828	262.32	20.14

**Table 4:** Results of the Integrated Model with Variable Sampling Intervals.

Failure Distribution	Parameters	Models	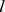	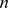	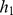	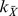	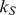	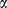	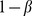	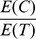	Reduction%
Weibull, Form = 1	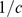 = 0.0505	BASE	-	38	3.69	1.91	1.34	0.0588	0.9875	239.62	-
		PM	0	37	5.33	1.89	1.33	0.0608	0.9864	219.88	8.24
		PM	1	41	5.72	1.89	1.27	0.0669	0.9917	244.43	−2.01
Weibull, Form = 1	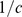 = 0.1010	BASE	-	35	2.68	1.81	1.36	0.0726	0.9844	320.16	-
		PM	0	36	3.56	1.76	1.36	0.0805	0.9866	291.24	9.03
		PM	1	42	4.26	1.79	1.26	0.0838	0.9935	319.47	0.22
Weibull, Form = 1	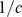 = 0.2525	BASE	-	34	1.56	1.71	1.35	0.0889	0.9844	473.33	-
		PM	0	33	2.32	1.63	1.32	0.1070	0.9845	426.32	9.93
		PM	1	40	2.78	1.71	1.27	0.0964	0.9926	449.60	5.01
Weibull, Form = 1	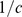 = 0.5050	BASE	-	30	1.14	1.60	1.31	0.1176	0.9796	618.24	-
		PM	0	32	1.77	1.59	1.31	0.1191	0.9838	560.70	9.31
		PM	1	42	2.45	1.53	1.25	0.1359	0.9953	561.57	9.17
Weibull, Form = 2	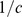 = 0.0505	BASE	-	37	7.56	1.89	1.38	0.0593	0.9858	214.93	-
		PM	0	40	10.77	1.91	1.34	0.0580	0.9898	196.39	8.63
		PM	1	42	11.72	2.08	1.26	0.0465	0.9909	221.90	−3.24
Weibull, Form = 2	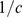 = 0.1010	BASE	-	33	4.62	1.69	1.43	0.0910	0.9815	300.85	-
		PM	0	34	7.06	1.70	1.36	0.0908	0.9845	269.92	10.28
		PM	1	37	7.62	1.84	1.35	0.0681	0.9870	299.98	0.29
Weibull, Form = 2	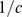 = 0.2525	BASE	-	30	2.66	1.59	1.41	0.1124	0.9778	461.88	-
		PM	0	31	3.96	1.61	1.37	0.1108	0.9805	410.79	11.06
		PM	1	38	4.82	1.62	1.24	0.1237	0.9920	430.66	6.76
Weibull, Form = 2	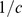 = 0.5050	BASE	-	33	1.86	1.64	1.27	0.1153	0.9853	617.15	-
		PM	0	30	2.67	1.59	1.41	0.1134	0.9778	546.43	11.46
		PM	1	37	3.35	1.52	1.29	0.1356	0.9916	539.53	12.58
Weibull, Form = 3	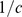 = 0.0505	BASE	-	38	10.55	1.94	1.34	0.0549	0.9871	199.31	-
		PM	0	46	11.57	2.19	1.37	0.0288	0.9922	194.30	2.51
		PM	1	42	11.44	1.95	1.34	0.0529	0.9914	220.49	−10.63
Weibull, Form = 3	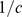 = 0.1010	BASE	-	33	6.44	1.73	1.49	0.0845	0.9796	282.51	-
		PM	0	36	9.04	1.83	1.42	0.0680	0.9846	251.94	10.82
		PM	1	38	10.12	1.93	1.26	0.0645	0.9882	279.06	1.22
Weibull, Form = 3	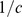 = 0.2525	BASE	-	31	3.32	1.60	1.46	0.1088	0.9785	441.29	-
		PM	0	35	4.79	1.66	1.36	0.0991	0.9867	388.99	11.85
		PM	1	36	5.72	1.57	1.21	0.1485	0.9909	401.09	9.11
Weibull, Form = 3	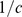 = 0.5050	BASE	-	31	1.99	1.50	1.46	0.1350	0.9811	593.08	-
		PM	0	31	3.11	1.49	1.37	0.1374	0.9827	519.36	12.43
		PM	1	38	3.77	1.57	1.27	0.1263	0.9922	508.15	14.32
Weibull, Form = 4	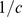 = 0.0505	BASE	-	41	11.42	2.04	1.44	0.0413	0.9881	191.11	-
		PM	0	42	11.48	2.00	1.51	0.0456	0.9889	203.49	−6.48
		PM	1	45	11.79	2.18	1.36	0.0295	0.9916	226.73	−18.64
Weibull, Form = 4	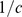 = 0.1010	BASE	-	30	7.40	1.60	1.38	0.1121	0.9782	268.60	-
		PM	0	40	10.71	2.00	1.33	0.0480	0.9889	240.34	10.52
		PM	1	40	11.38	1.93	1.29	0.0588	0.9901	262.89	2.13
Weibull, Form = 4	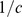 = 0.2525	BASE	-	31	3.49	1.61	1.39	0.1097	0.9801	423.42	-
		PM	0	32	5.37	1.62	1.31	0.1120	0.9832	367.06	13.31
		PM	1	36	5.97	1.59	1.28	0.1203	0.9898	378.43	10.63
Weibull, Form = 4	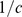 = 0.5050	BASE	-	31	2.01	1.50	1.33	0.1384	0.9833	575.26	-
		PM	0	30	3.12	1.59	1.31	0.1194	0.9798	495.23	13.91
		PM	1	40	3.95	1.52	1.30	0.1314	0.9938	484.94	15.70
Gamma, Form = 2	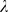 = 0.0505	BASE	-	43	11.98	2.03	1.39	0.0426	0.9909	182.60	-
		PM	0	52	11.72	2.46	1.51	0.0139	0.9931	189.32	−3.68
		PM	1	52	11.75	2.46	1.36	0.0142	0.9943	202.43	−10.86
Gamma, Form = 2	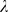 = 0.1010	BASE	-	35	8.72	1.80	1.35	0.0735	0.9845	239.97	-
		PM	0	41	11.56	2.00	1.42	0.0452	0.9888	219.11	8.69
		PM	1	42	10.58	2.04	1.40	0.0418	0.9897	232.71	3.03
Gamma, Form = 2	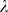 = 0.2525	BASE	-	35	5.11	1.75	1.40	0.0802	0.9845	359.85	-
		PM	0	35	6.47	1.81	1.43	0.0701	0.9829	323.11	10.21
		PM	1	35	5.73	1.81	1.44	0.0698	0.9828	326.87	9.16
Gamma, Form = 2	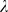 = 0.5050	BASE	-	32	3.38	1.62	1.52	0.1059	0.9794	478.37	-
		PM	0	36	4.33	1.73	1.42	0.0836	0.9860	433.11	9.46
		PM	1	39	3.72	1.98	1.31	0.0510	0.9882	417.41	12.74

#### Constant Sampling Intervals

The results of the tests with constant sampling intervals are presented in Table 3, where BASE represents the solution of the base cost function model (Eq. 27 and 30) applied for the ESD of 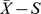 control charts. PM represents the integrated cost function model (Eq. 52 and 54) with 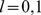.

In Table 3 for the Exponential distribution with 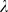 = 0.0505 there are reductions (savings) in the costs (3.73% and 1.12%) when PM is implemented without interruption (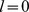) or with interruption (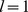) of the process. These reductions are higher when the failure rates increase: 5.47% and 7.64% for 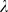 = 0.1010; 6.79% and 16.08% for 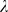 = 0.2525; and 6.74% and 21.93% for 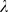 = 0.5050.

For the Weibull distribution, when the failure rate is small (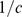 = 0.0505) and 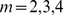, small or no reductions are obtained when PM is performed without interruption of the process: 1.88%, 0.30%, and −0.93% respectively. Reductions are obtained when the failure rate increases to 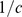 = 0.1010: 3.67%, 1.36%, and 0.91% respectively. However, if PM is performed with interruption of the process for 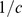 = 0.0505 and 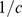 = 0.1010, the costs are higher than the baseline (BASE) and negative reductions are obtained.

On the other hand, when the failure rate increases to 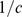 = 0.2525 and 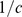 = 0.5050 the reductions are consistently high and positive for both scenarios (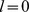, 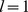). For example, for 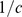 = 0.5050 and 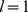 the reductions are 25.92%, 20.83%, and 15.37% for 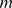 = 2,3,4. In just one case, when 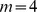 and 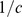 = 0.2525, a negative reduction was obtained (−0.95%).

A similar pattern is observed for the Gamma distribution, where there are reductions when the failure rate is small and the process is not interrupted during PM: 2.11% for 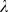 = 0.0505 and 4.24% for 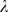 = 0.1010. Negative reductions are obtained when the process is interrupted with the same failure rates: −7.31% and 2.86% respectively. Consistent reductions are obtained when 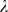 = 0.2525 and 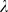 = 0.5050: 7.13% and 9.65% when 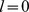, and 8.30% and 20.14% when 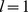 respectively. Note than in all cases with PM the length of the sampling interval (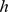) was increased.

A paired Student's t-Test was performed to determine the statistical significance of the results presented in Table 3. The overall reduction obtained with 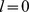 was significant with a 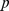-value of 0.000035481 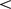 0.05, 0.01. For 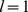 the reduction was significant with a 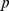-value of 0.003052452 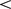 0.05, 0.01.

**Figure 13:**
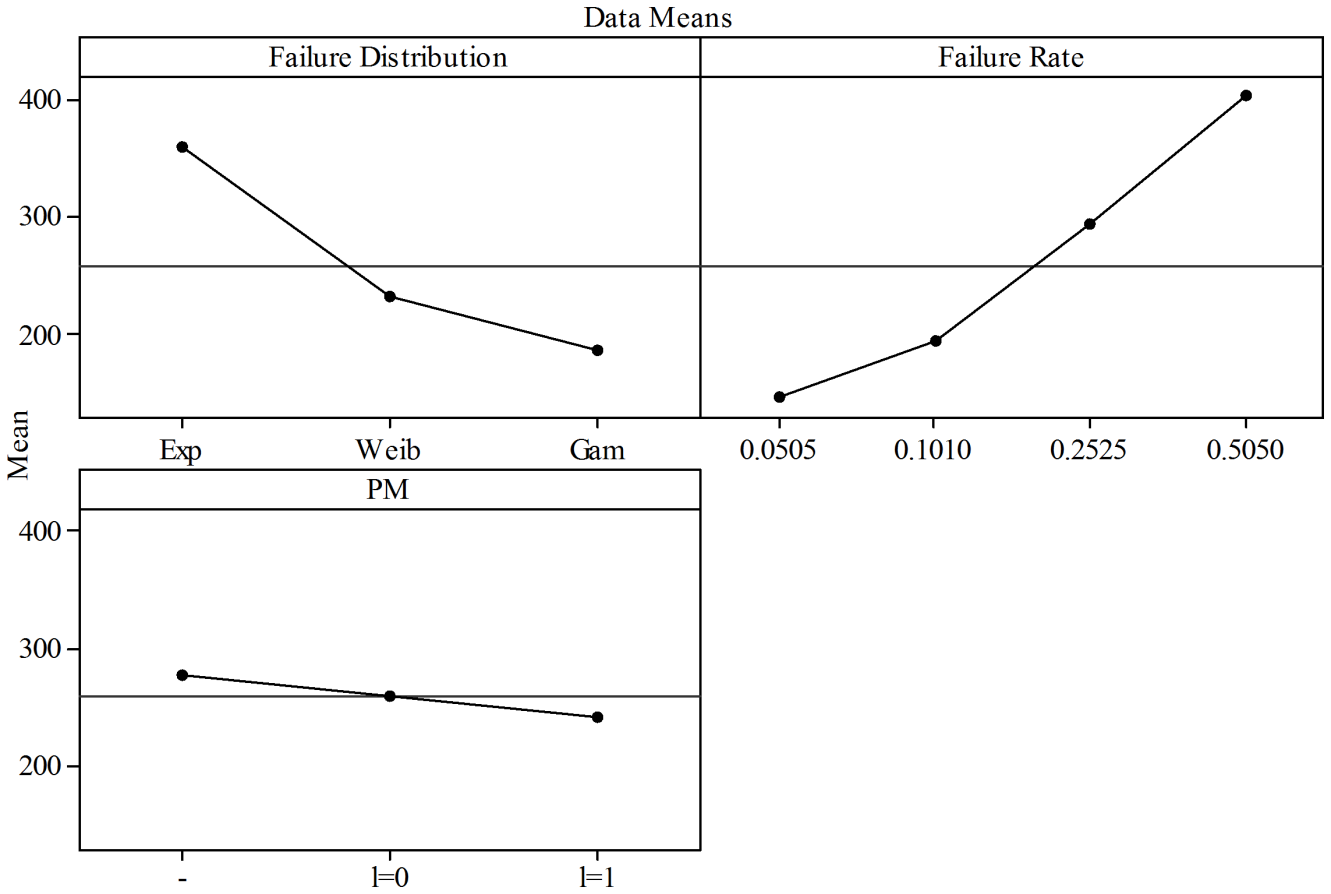
Main Effects Plots for 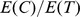 and Constant Sampling Intervals - All Failure Distributions Three main factors were considered: Failure distributions (three levels: Exponential, Weibull, and Gamma), Failure rate (four levels: 0.0505, 0.1010, 0.2525, and 0.5050), and PM (three levels: no implementation -, implementation with 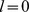, and implementation with 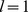). The first plot shows that overall costs decrease based on the failure distribution used to model the failure behavior. The cost model with Exponential distribution has the higher costs (given by the ratio 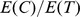) while the Gamma has the lowest costs considering all the other factors. The second plot shows that as failure rate increases from 0.0505 to 0.5050 the cost increases considering the other factors. The third plot shows that, considering failure distributions and failure rates, PM is responsible for decreasing costs from the base model where no PM is performed (-). The maximum reduction is achieved when PM is performed with interruption of the production process (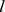 = 1).

A factorial analysis was performed on the data presented in Table 3 to assess the effect of PM on the overall cost reductions when considered with the other factors in the cost models. Minitab ver.15.1.30.0. was used for this purpose and in Figure 13 the Main Effects Plots for 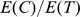 are presented. Three main factors were considered:

1. Failure distributions. Three levels: Exponential, Weibull, and Gamma. For this analysis an 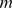 = 2 was used for the Weibull distribution.
2. Failure rate. Four levels: 0.0505, 0.1010, 0.2525, and 0.5050.
3. PM. Three levels: no implementation  =  BASE (-), implementation with 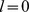, and implementation with 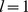.

The first plot shows that overall costs decrease based on the failure distribution used to model the failure behavior. The cost model with Exponential distribution has the higher costs (given by the ratio 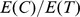) while the Gamma has the lowest costs considering all the other factors (failure rate and PM). The second plot shows that as failure rate increases from 0.0505 to 0.5050 the cost increases considering the other factors (failure distribution and PM). The third plot shows that, considering failure distributions and failure rates, PM is responsible for decreasing costs from the base model where no PM is performed (-). The maximum reduction is achieved when PM is performed with interruption of the production process (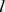 = 1).

**Figure 14:**
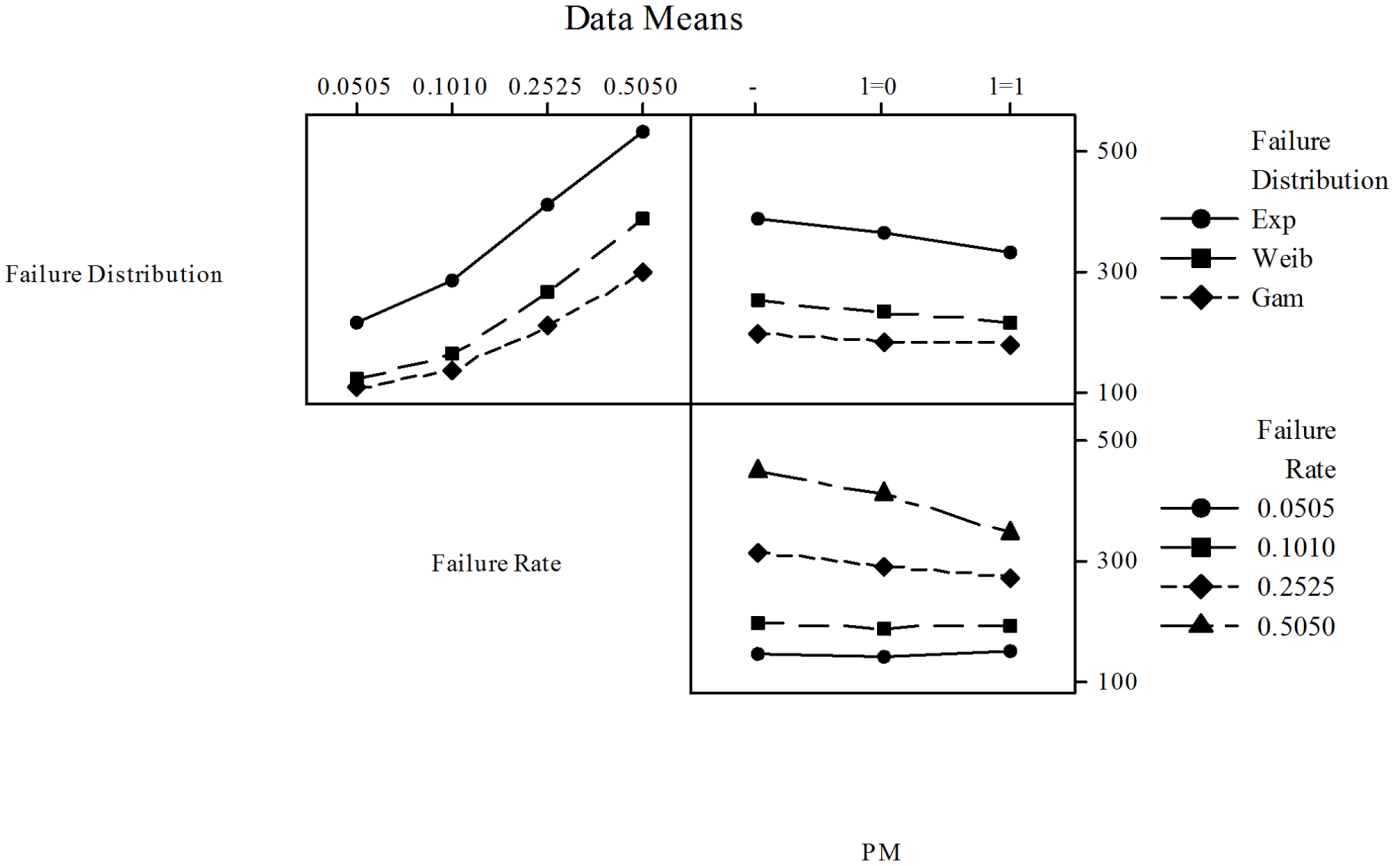
Interaction Plots for 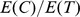 and Constant Sampling Intervals - All Failure Distributions. Three main interactions are considered: (1) Failure Distribution vs. Failure Rate - All costs increase as failure rate increases. Costs are the highest for the Exponential distribution, and the lowest for the Gamma distribution; (2) Failure Distribution vs. PM - PM decreases costs for the Exponential, Weibull, and Gamma distributions; (3) Failure Rate vs. PM - In general, if the failure rate is small (0.0505, 0.1010) there are no savings or cost reductions. As the failure rate increases the savings become more significant when PM is performed.

In Figure 14 the Interaction Plots for 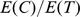 are presented and the following is observed:

- Failure Distribution vs. Failure Rate. All costs increase as failure rate increases. Costs are the highest for the Exponential distribution, and the lowest for the Gamma distribution.
- Failure Distribution vs. PM. PM decreases costs for the Exponential, Weibull, and Gamma distributions.
- Failure Rate vs. PM. In overall, if the failure rate is small (0.0505, 0.1010) there are no savings or cost reductions. As the failure rate increases the cost reductions are more evident when PM is performed.

**Figure 15:**
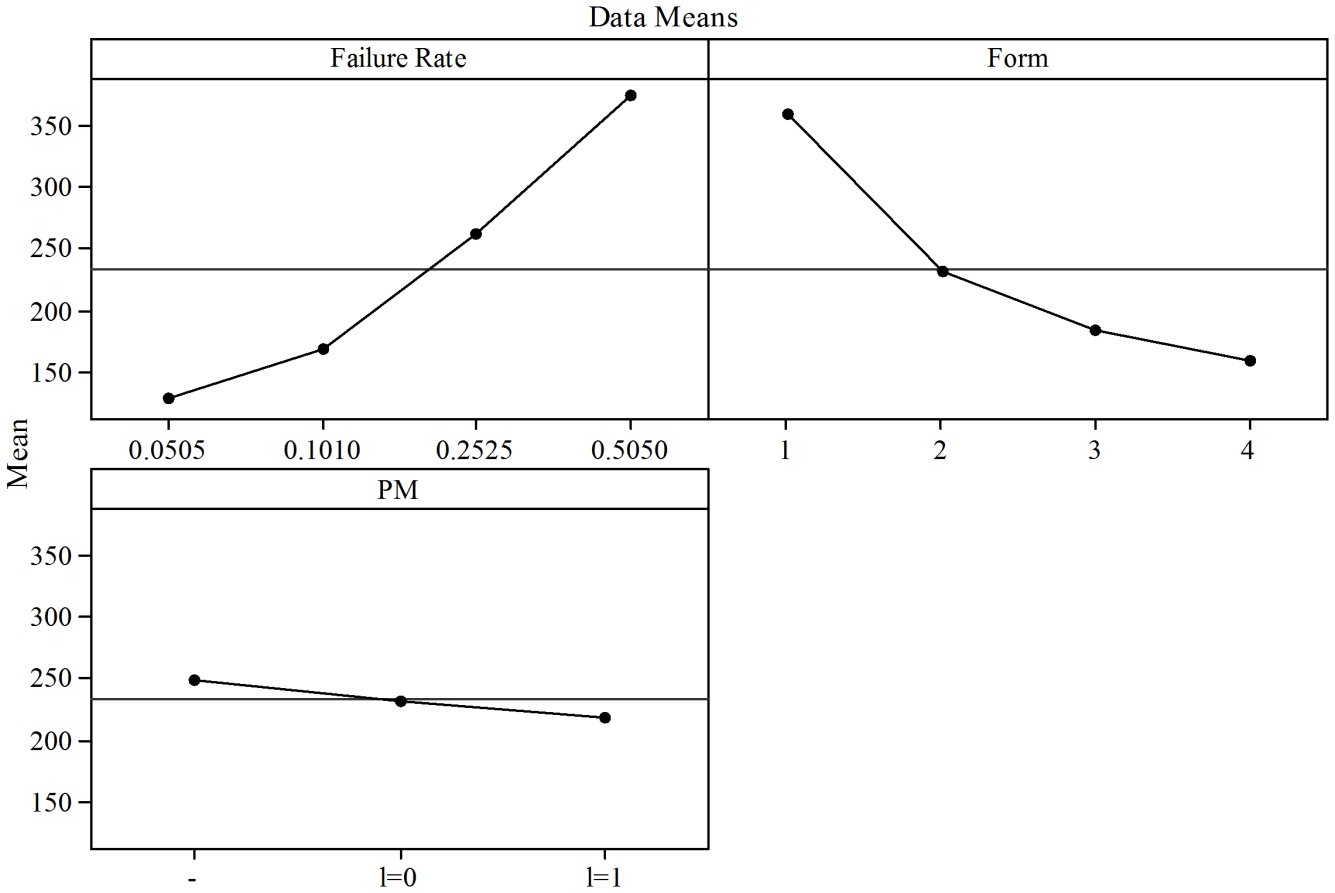
Main Effects Plots for 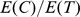 and Constant Sampling Intervals - Weibull Distribution. Three main factors were considered: Failure rate (four levels: 0.0505, 0.1010, 0.2525, and 0.5050), Form parameter (four levels: 1, 2, 3, 4), and PM (three levels: no implementation -, implementation with 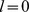, and implementation with 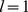). As presented, the cost ratio 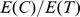 increases as the failure rate does. However there is an inverse relationship between the 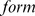 parameter and the cost given by 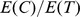. Considering the failure rate and the 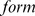 parameter, performing PM decreases the ratio 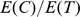.

**Figure 16:**
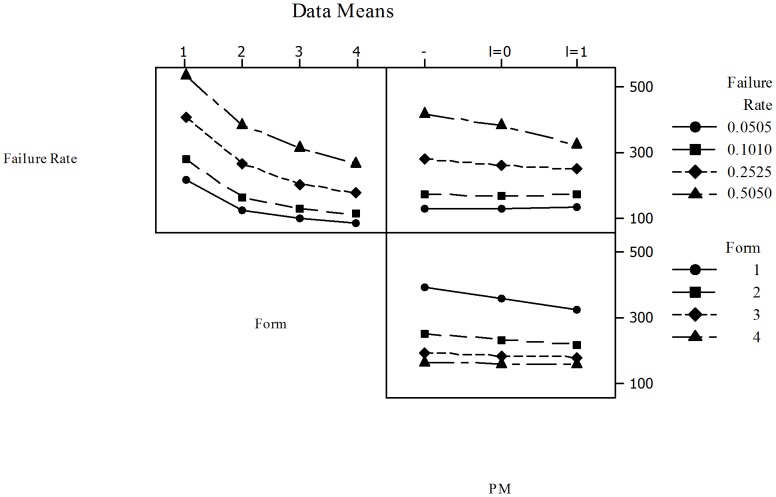
Interaction Plots for 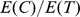 and Constant Sampling Intervals - Weibull Distribution. Three main interactions were considered: (1) Failure Rate vs. Form parameter - As 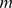 increases the cost ratio 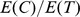 decreases for all failure rates although all costs increase as failure rate increases; (2) Failure Rate vs. PM - Performing PM has no significant effect on cost reduction for small failure rates (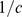 = 0.0505, 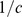 = 0.1010). Reductions are achieved for higher failure rates as 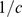 = 0.2525 and 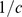 = 0.5050; (3) Form vs. PM - There is no significant difference in cost when 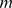 varies from 3 to 4 and thus no relationship between PM and the 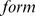 parameter is evident. When 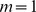 the most significant reduction is achieved, however this is the case where the Weibull distribution is approximated to the Exponential. When 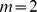 the reduction is less evident.

The same analysis was performed for the results obtained with the Weibull distribution. This was performed to assess the effect of PM when considered with the 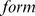 parameter of the failure distribution. In Figures 15 and 16 the Main Effects Plots and the Interaction Plots for 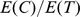 are presented. As presented in Figure 15 the cost increases as the failure rate does. However there is an inverse relationship between the 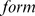 parameter and the cost given by 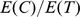. Considering the failure rate and the 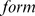 parameter, performing PM decreases the ratio 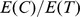.

When analyzing the interaction plots (Figure 16) it is observed that as 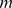 increases the cost decreases for all failure rates. Performing PM has no significant effect on cost reduction for small failure rates (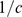 = 0.0505, 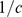 = 0.1010). Reductions are achieved for higher failure rates as 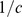 = 0.2525 and 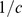 = 0.5050. There is no noticeable difference in cost when 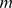 varies from 3 to 4 and thus no relationship between PM and the 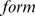 parameter of the Weibull distribution is evident. When 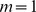 the highest reduction is achieved, however this is the case where the Weibull distribution is approximated to the Exponential. When 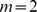 the reduction is less evident.

Thus, the 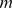 parameter of the Weibull distribution has no significant effect on the performance of PM. Also, for all distributions with failure rates over 0.15 the PM generates reductions in 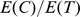.

#### Variable Sampling Intervals

The results of the tests with variable sampling intervals are presented in Table 4, where BASE represents the solution of the base cost function model (Eq. 37 and 50) applied for the ESD of 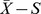 control charts. PM represents the integrated cost function model (Eq. 58 and 59). As presented in Table 4, Weibull and Gamma distributions with small failure rate (0.0505) have some instances where the reductions in costs are very small or even negative when PM is implemented with 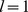. For higher failure rates the reductions increase to approximately 10% of the BASE model when 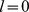.

In general, the results presented in Table 4 for PM with 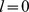 were statistically significant with a 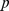-value of 0.0000033257 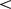 0.05, 0.01. For PM with 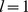, the results were significant with a 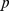-value of 0.0096247835 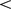 0.05, 0.01.

**Figure 17:**
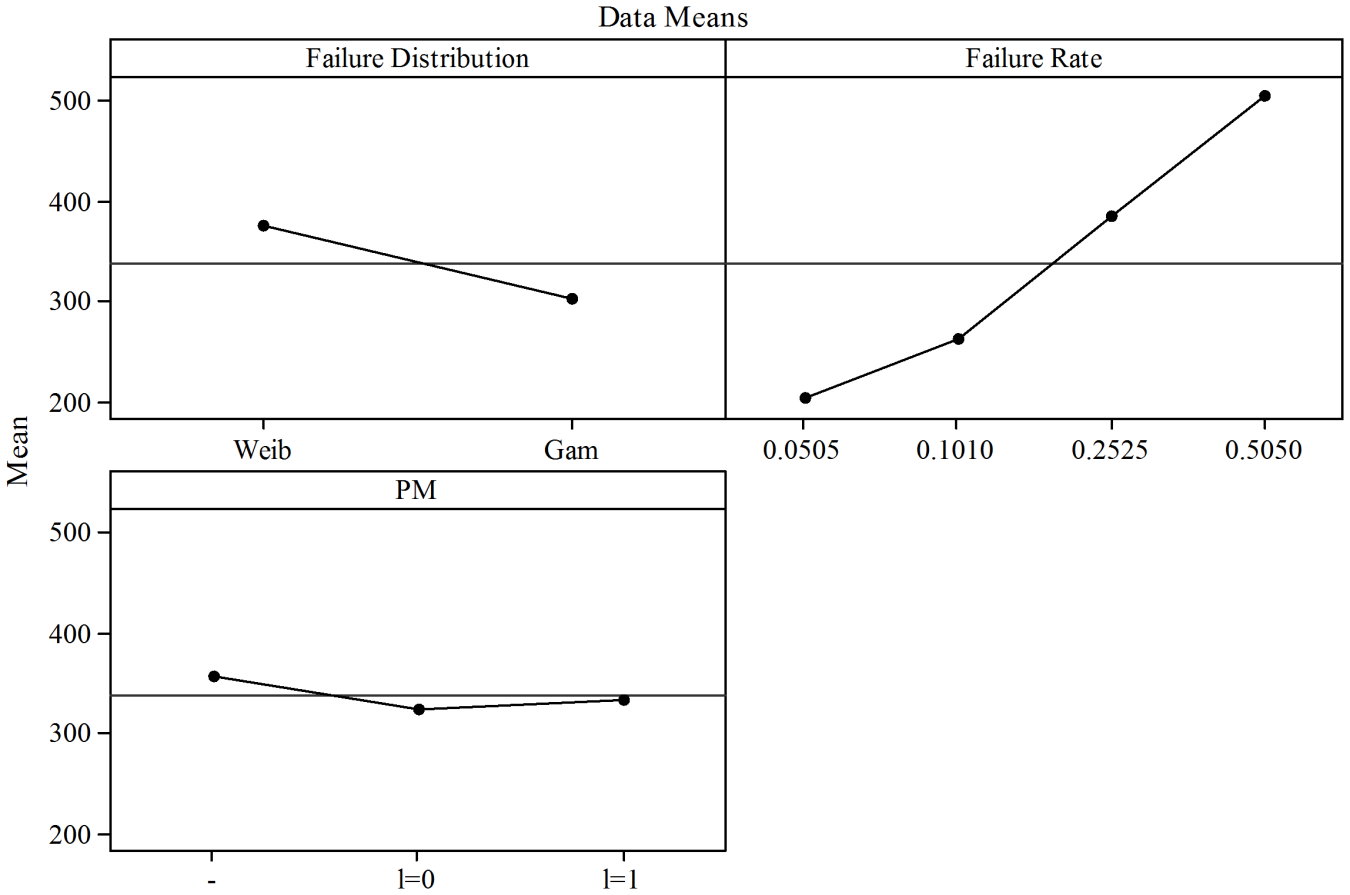
Main Effects Plots for 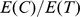 and Variable Sampling Intervals - All Failure Distributions Two main factors were considered: Failure distributions (two levels: Weibull and Gamma), Failure rate (four levels: 0.0505, 0.1010, 0.2525, and 0.5050), and PM (three levels: no implementation -, implementation with 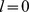, and implementation with 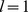). The first plot shows that overall costs decrease based on the failure distribution used to model the failure behavior. The cost model with Weibull distribution has the highest costs (given by the ratio 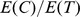) while the Gamma has the lowest costs considering all the other factors. The second plot shows that as failure rate increases from 0.0505 to 0.5050 the cost increases considering the other factors. The third plot shows that, considering failure distributions and failure rates, PM is responsible for decreasing costs from the base model where no PM is performed (-). In contrast with constant sampling intervals, when failure rate is within 0.1010 and 0.2525 the most significant reduction is achieved when PM is performed without interruption of the production process (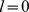).

In the Main Effects Plot of Figure 17 is observed that the cost model with Gamma distribution has a lower ratio 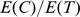 than the Weibull distribution. Also, in general terms, the ratio increases as the failure rate does. Note however that, in comparison with constant sampling intervals, when failure rate is within 0.1010 and 0.2525 the highest reduction is achieved when PM is performed without interruption of the production process (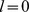). In addition, the length of the sampling intervals is increased (in this case, starting from 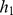).

**Figure 18:**
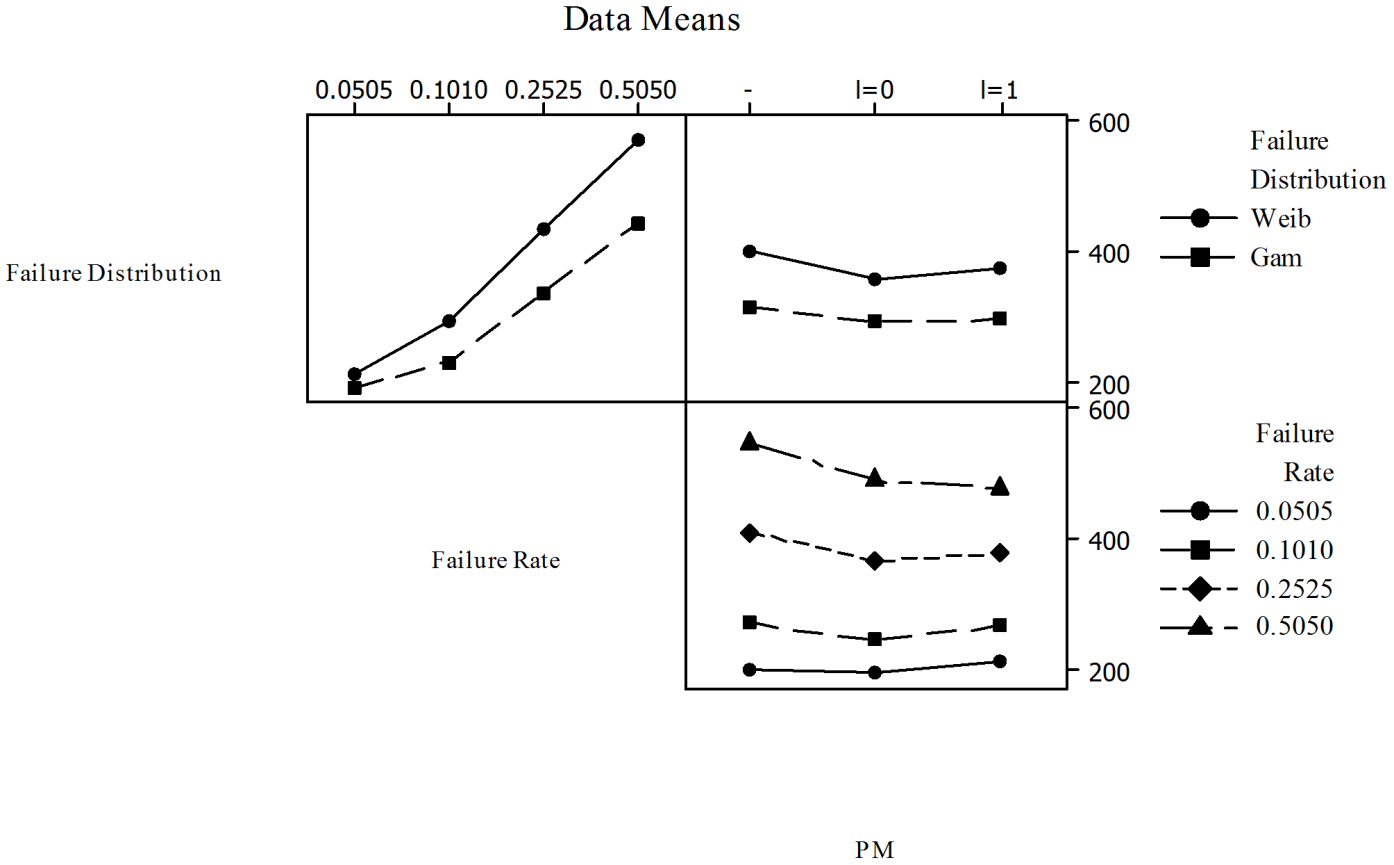
Interaction Plots for 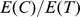 and Variable Sampling Intervals - All Failure Distributions. Three main interactions were considered: (1) Failure Distribution vs. Failure Rate - All costs increase as failure rate increases. Costs are higher for the Weibull distribution; (2) Failure Distribution vs. PM - PM decreases costs for the Weibull and Gamma distributions, being the most significant for the model with Weibull distribution. For the Gamma distribution there is no observable difference in the performance of PM with or without interruption in the production process; (3) Failure Rate vs. PM - In general, if the failure rate is small (0.0505) there are no savings or cost reductions. As the failure rate increases the savings become more significant when PM is performed. For failure rates of 0.1010 and 0.2525 the highest reduction is obtained when PM is performed without interruption of the process. However, for the highest failure rate (0.5050) the maximum reduction is achieved when PM is performed with interruption as observed in the case of constant sampling intervals.

In Figure 18 the Interaction Plots for 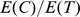 are presented and the following is observed:

- Failure Distribution vs. Failure Rate. All costs increase as failure rate increases. Costs are higher for the Weibull distribution.
- Failure Distribution vs. PM. PM decreases costs for the Weibull and Gamma distributions, being the highest for the model with Weibull distribution. For the Gamma distribution there is no observable difference in the performance of PM with or without interruption in the production process.
- Failure Rate vs. PM. In overall, if the failure rate is small (0.0505) there are no savings or cost reductions. As the failure rate increases the savings increase when PM is performed. For failure rates of 0.1010 and 0.2525 the highest reduction is obtained when PM is performed without interruption of the process. However, for the highest failure rate (0.5050) the maximum reduction is achieved when PM is performed with interruption as observed in the case of constant sampling intervals.

The TS algorithm and the estimation of parameters led to lower levels of 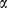 and 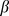 than those specified for the restrictions in the ESD (see Table 2). This is achieved for constant and variable sampling cost models with all failure distributions.

## Conclusions

The deduction of the models of Rahim *et al.* [21], [28] and the adaptation to incorporate PM in the renewal equations can be used for future research or adaptation to other control charts. The ESD of 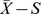 considering these cost models is important because a joint control chart with the same values for 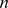 and 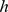 can monitor both, the mean and the variability, of the quality characteristic of the process, thus keeping a better SPC. By keeping also control on the probabilities of the errors Type I and II, the presented ESD can provide parameters that would lead to control charts with low rates of false alarms (error Type I, unnecessary interruptions), and low production of faulty products (prompt detection).

From the results presented in Tables 3 and 4 it was observed that, when the failure rates were small, there was little or no cost benefit in performing PM with different failure probability distributions. This can be attributed to the concept that a “good” process does not need much maintenance as a “bad” process (with higher failure rates) would require. In the case of high failure rates it is convenient to perform PM with significant cost benefits, either with or without interruption of the process. A result of the effect of PM on the reliability of the process would be the increase in the length of the sampling intervals (constant or variable), which means a reduction in the sampling frequency. The results presented in this paper corroborate these findings.

Future work is focused on: (1) modelling techniques or methods for the gain 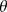 obtained by performing PM; (2) incorporate multiple assignable causes in the integrated cost function model with PM; (3) consider other probability failure distributions and cost models; and (4) integrate PM on the ESD of specific control charts as EWMA and CUSUM for the detection of small shifts (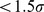).
